# Patterns of Immune Infiltration in Breast Cancer and Their Clinical Implications: A Gene-Expression-Based Retrospective Study

**DOI:** 10.1371/journal.pmed.1002194

**Published:** 2016-12-13

**Authors:** H. Raza Ali, Leon Chlon, Paul D. P. Pharoah, Florian Markowetz, Carlos Caldas

**Affiliations:** 1 Cancer Research UK Cambridge Institute, University of Cambridge, Cambridge, United Kingdom; 2 Department of Pathology, University of Cambridge, Cambridge, United Kingdom; 3 Cambridge Experimental Cancer Medicine Centre and NIHR Cambridge Biomedical Research Centre, Cambridge, United Kingdom; 4 CRUK & EPSRC Cancer Imaging Centre in Cambridge and Manchester, Cambridge, United Kingdom; 5 Department of Oncology, University of Cambridge, Cambridge, United Kingdom; MSKCC, UNITED STATES

## Abstract

**Background:**

Immune infiltration of breast tumours is associated with clinical outcome. However, past work has not accounted for the diversity of functionally distinct cell types that make up the immune response. The aim of this study was to determine whether differences in the cellular composition of the immune infiltrate in breast tumours influence survival and treatment response, and whether these effects differ by molecular subtype.

**Methods and Findings:**

We applied an established computational approach (CIBERSORT) to bulk gene expression profiles of almost 11,000 tumours to infer the proportions of 22 subsets of immune cells. We investigated associations between each cell type and survival and response to chemotherapy, modelling cellular proportions as quartiles. We found that tumours with little or no immune infiltration were associated with different survival patterns according to oestrogen receptor (ER) status. In ER-negative disease, tumours lacking immune infiltration were associated with the poorest prognosis, whereas in ER-positive disease, they were associated with intermediate prognosis. Of the cell subsets investigated, T regulatory cells and M0 and M2 macrophages emerged as the most strongly associated with poor outcome, regardless of ER status. Among ER-negative tumours, CD8+ T cells (hazard ratio [HR] = 0.89, 95% CI 0.80–0.98; *p* = 0.02) and activated memory T cells (HR 0.88, 95% CI 0.80–0.97; *p* = 0.01) were associated with favourable outcome. T follicular helper cells (odds ratio [OR] = 1.34, 95% CI 1.14–1.57; *p* < 0.001) and memory B cells (OR = 1.18, 95% CI 1.0–1.39; *p* = 0.04) were associated with pathological complete response to neoadjuvant chemotherapy in ER-negative disease, suggesting a role for humoral immunity in mediating response to cytotoxic therapy. Unsupervised clustering analysis using immune cell proportions revealed eight subgroups of tumours, largely defined by the balance between M0, M1, and M2 macrophages, with distinct survival patterns by ER status and associations with patient age at diagnosis. The main limitations of this study are the use of diverse platforms for measuring gene expression, including some not previously used with CIBERSORT, and the combined analysis of different forms of follow-up across studies.

**Conclusions:**

Large differences in the cellular composition of the immune infiltrate in breast tumours appear to exist, and these differences are likely to be important determinants of both prognosis and response to treatment. In particular, macrophages emerge as a possible target for novel therapies. Detailed analysis of the cellular immune response in tumours has the potential to enhance clinical prediction and to identify candidates for immunotherapy.

## Abstract 2

To investigate tumor infiltration by different types of immune cells, H. Raza Ali and colleagues study gene expression profiles from large breast cancer datasets.

## Author Summary

**Why Was This Study Done?:**

- Previous studies have shown that certain immune cells present in breast tumours are associated with risk of relapse.
- Whether particular immune cell types are associated with a greater or lesser risk of relapse, however, and how these effects differ by breast cancer subtype, remains unclear.

**What Did the Researchers Do and Find?:**

- We conducted a large analysis of breast tumour gene expression profiles available in the public domain (10,988 cases) to derive estimates of the relative proportions of 22 subsets of immune cells, in order to investigate associations between the proportion of each cell type and disease relapse or response to chemotherapy.
- We found that higher proportions of some immune cell types were associated with greater risk of relapse (or greater chemotherapy response), whereas others were associated with lesser risk, and that these associations were often different according to the oestrogen receptor (ER) status of the tumour.
- In tumours lacking expression of ER, we found that the presence of CD8+ T cells and activated memory T cells was associated with a reduction in the risk of relapse, while tumours with high proportions of T follicular helper cells were more likely to respond to neoadjuvant chemotherapy.
- In ER-positive tumours, the presence of M0 macrophages was associated with poor prognosis.
- T regulatory cells were associated with poor prognosis in both ER-positive and ER-negative tumours.

**What Do These Findings Mean?:**

- These findings establish a complex relationship between the heterogeneity of intratumoural immune cells, tumour molecular subtype, and disease progression in breast cancer.
- Treatments that aim to boost the immune response to tumours, i.e., immunotherapies, are effective in only a subset of patients, and our findings may help to identify this patient group and suggest targets for the development of new immunotherapies.

## Introduction

Breast cancer is characterised by biological and clinical diversity. Genomic changes in cancer cells have been extensively investigated to identify patient subgroups with different prognoses and different responses to treatment, as well as to find new drug targets [1–3]. However, breast tumours are composed of intimate mixtures of cancer cells and non-cancer cells. The roles of these non-cancer cells remain poorly understood. Non-cancer cells compose varying proportions of tumours and include stromal cells, vascular cells, and infiltrating immune cells. Of these, infiltrating immune cells seem the most likely to improve the prediction of clinical outcome and to be effectively targeted by drugs. This is because recent trials of drugs that target immune checkpoints [4–7]—physiological pathways that regulate the immune response to self as well as immune response duration and severity in disease—have shown that they can significantly prolong the survival of a subset of patients with solid tumours including melanoma, non-small-cell lung carcinoma, renal cell carcinoma [5], and, most recently, triple-negative breast cancer [6]. Also, past studies have shown that the immune response is associated with both clinical outcome and response to treatment in breast cancer [8–13]. Owing to technical limitations, these studies have necessarily been limited to a very narrow view of the immune response, generally including only one or two cell types. The immune response, however, is characterised by numerous specialised cell types that interact in a highly coordinated manner. To better understand the nature and diversity of the immune response to breast tumours, it is therefore necessary to enumerate immune cells in a way that accounts for the breadth of their specialised functions. In addition, to reliably investigate the interaction of the immune response with different molecular subtypes of breast cancer, it is also necessary to analyse very large cohorts of patients encompassing the molecular diversity of the disease.

The aim of this study was to quantify the cellular composition of the immune response in breast tumours in order to investigate its relationship with molecular subtype, survival, and response to chemotherapy. We applied a recently described gene expression deconvolution algorithm (CIBERSORT) [14], which estimates the relative proportions of 22 distinct functional subsets of immune cells, to almost 11,000 breast tumour transcriptomes with clinical annotation and data in the public domain.

## Methods

### Gene Expression Datasets

**Fig 1:**
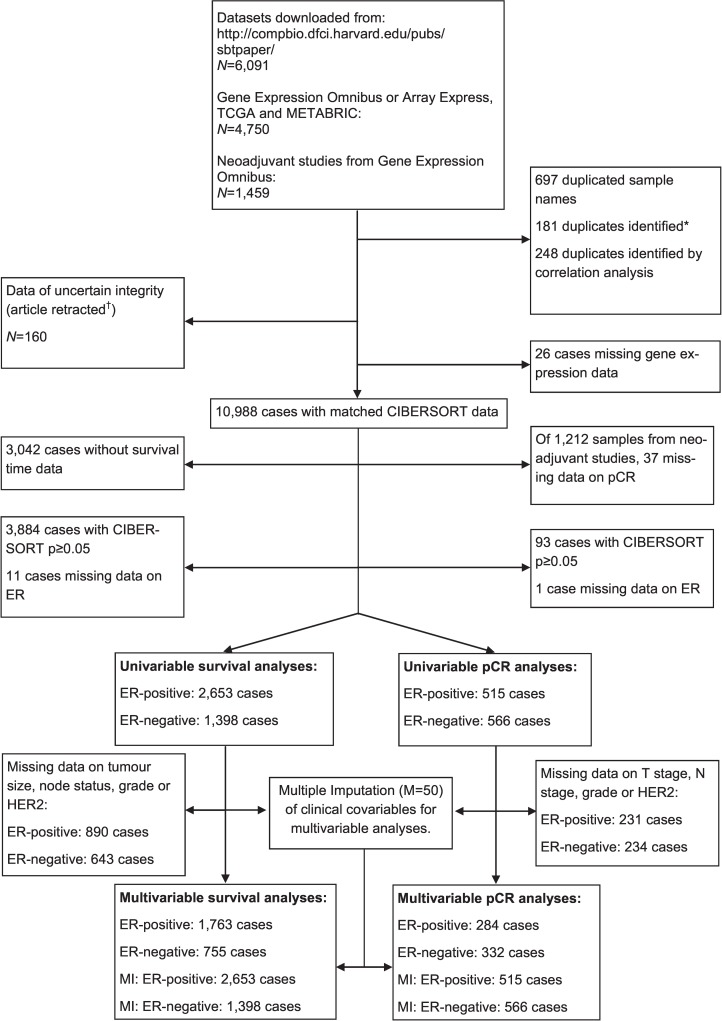
Study flowchart detailing the flow of samples at each stage of analysis. *[15]. ^†^[16]. ER, oestrogen receptor; pCR, pathological complete response; MI, multiple imputation; TGCA, The Cancer Genome Atlas.

This study made use of data in the public domain. Details of ethical approval and patient consent for all 56 studies can be found in their corresponding publications specified in S1 Table. In total, 56 studies were included, of which 32 were compiled and curated by Haibe-Kains et al. (data available at http://compbio.dfci.harvard.edu/pubs/sbtpaper/) as previously described [15]. Additional studies were downloaded from Gene Expression Omnibus and ArrayExpress. Data from The Cancer Genome Atlas (TCGA) were downloaded from the TCGA data portal in April 2016. All studies with gene expression data from primary breast tumours were considered eligible, with no specific exclusion criteria applied. One study was removed because the article had since been retracted [16]. Studies are summarised, together with accession numbers, in S1 Table. Cases identified as replicates by Haibe-Kains et al. were removed (*n* = 181). Samples with the same accession identifier were also removed (*n* = 697). Further replicates were identified by computing Pearson correlations between all samples arising from the same centre. Samples with Pearson correlations of 0.99 or greater were considered replicates (*n* = 248). Where replicate cases were identified, all but one record were removed from further analysis. In total, 10,988 cases were available for analysis following removal of replicate records. Details of which samples were included at each stage of analysis are illustrated in Fig 1 as a flowchart. Each tumour sample corresponded to one patient. No protocol or prospective analysis plan was specified for the study.

### Molecular Subtyping and Inference of Infiltrating Immune Cells

Oestrogen receptor (ER) and human epidermal growth factor receptor 2 (HER2) status were preferentially determined by study annotation. Where unavailable, normalised gene expression data were used to infer probable ER or HER2 status using a two-component Gaussian finite mixture model using maximum likelihood estimation on a per-study basis, in a manner similar to that previously described [17]. Where more than one probe was available for either gene, the probe associated with the highest log-likelihood from the mixture regression model was used. Whether inferred receptor status was considered sufficiently reliable for analysis was determined by examining histograms of the distribution of expression values and the relative proportions of inferred positive and negative tumours. Where the distribution of expression data clearly departed from two Gaussian distributions or where inferred proportions of positive and negative status were judged implausible based on study annotation, inferred ER and HER2 status was not used in further analysis.

Classification into the ten IntClust molecular subtypes was achieved based on gene expression data using the iC10 package [18] in R, as previously described [2]. Distributions of molecular and clinical characteristics by study are detailed in S2 Table.

Normalised gene expression data were used to infer the relative proportions of 22 types of infiltrating immune cells using the CIBERSORT algorithm. For the TCGA dataset [3], RNA sequencing data were transformed using voom [19] (variance modelling at the observational level), converting count data to values more similar to those resulting from microarrays. The 22 cell types inferred by CIBERSORT encompass B cells, T cells, natural killer cells, macrophages, dendritic cells, eosinophils, and neutrophils, amongst others. CIBERSORT is a deconvolution algorithm that uses a set of reference gene expression values (a “signature matrix” of 547 genes) considered a minimal representation for each cell type and, based on those values, infers cell type proportions in data from bulk tumour samples of mixed cell types using support vector regression [14]. CIBERSORT derives a *p*-value for the deconvolution for each sample using Monte Carlo sampling, providing a measure of confidence in the results. Gene expression datasets were prepared using standard annotation files and data uploaded to the CIBERSORT web portal (http://cibersort.stanford.edu/), with the algorithm run using the default signature matrix at 1,000 permutations. Since we pooled numerous studies published over some years, the technologies used to measure gene expression differed substantially, with variable representation of the 547 genes that comprise the signature matrix. In order to estimate the effect of this variable representation, we used 100 cases from the METABRIC study, where 87% of the genes are represented. We randomly deleted signature matrix genes in increments of 10% (55 genes) until 7% of genes remained to produce a graded representation of signature matrix genes. We ran CIBERSORT across these conditions and compared the output. Immune cytolytic activity representing the geometric mean of *GZMA* and *PRF1* is another in silico measure of immune infiltration, as described by Rooney et al. [20]. Immune cytolytic activity for the TCGA dataset was used, as provided in the supplement of Rooney et al. [20], and was calculated for the METABRIC study as the geometric mean of *GZMK* and *PRF1*.

### Statistical Analyses

Associations between inferred proportions of immune cell types and survival were tested using Cox regression. Analyses were conducted separately for ER-negative and ER-positive disease to account for this fundamental difference in breast cancer and for known violations of the Cox proportional-hazards assumption [21]. Cox regression analyses were stratified by study, with the five contributing centres of the METABRIC [1] study considered separate strata. Although the diversity of studies included meant that different types of follow-up were recorded, a single survival time variable was generated to enable derivation of more precise estimates owing to larger sample size. Where more than one type of follow-up was available, the following hierarchy was used to select one: (1) relapse-free survival, (2) distant metastasis-free survival, (3) disease-specific survival, and (4) overall survival. The type of survival time available for each study is detailed in S1 Table. Survival analyses were truncated at 15 y. Cases with a CIBERSORT *p*-value of <0.05 were included in the main survival analysis. Quartiles of the proportion of each cell type were computed for survival analysis and modelled as continuous variables in order to derive more easily interpretable hazard ratios (HRs). Quartiles were calculated separately by ER status or relevant subgroup. Where all samples were included in analyses irrespective of CIBERSORT *p*-value, cases with an empirical CIBERSORT *p ≥* 0.05 formed a separate baseline reference category to which the quartiles of proportion, based on cases with an empirical CIBERSORT *p <* 0.05, were compared, yielding five groups in total (0: all cases with a CIBERSORT *p ≥* 0.05, 1–4: quartiles of immune cell proportion for remaining cases with a CIBERSORT *p <* 0.05). Heterogeneity of estimates in subgroup analyses was tested using Cochran’s Q test. Where subgroup analyses comprised both ER-positive and ER-negative tumours, models were adjusted for ER status and an interaction term allowed to vary as a function of the logarithm of time included, accounting for violations of the proportional-hazards assumption. Interaction terms between ER and cell type proportion were included in Cox regression models to evaluate differences in effect by ER status. Immune cell subsets significantly associated with outcome in unadjusted analyses were included in multivariable models. Multivariable analyses were adjusted for lymph node status (negative, positive), tumour size (<10 mm, 10–19 mm, 20–29 mm, 30–49 mm, 50+ mm), histological grade, and HER2 status. Ordinal categories of tumour size and histological grade were modelled as continuous variables. The log-rank statistic was used to assess differences in survival between groups as depicted in Kaplan-Meier survival plots. Analyses complied with STROBE criteria [22]. A STROBE checklist is provided as S3 Table.

To assess the association between different immune cell types and response to chemotherapy, we used data from studies of neoadjuvant chemotherapy (where patients received chemotherapy before surgery). The endpoint of these studies was pathological complete response (pCR). Although there is some variation in the definition of pCR, it usually refers to the complete elimination of cancer cells, as assessed by careful histology of the primary tumour bed following surgery. Associations between different immune cell types and pCR were assessed by logistic regression. Quartiles of proportions of each immune cell type were modelled as continuous variables. Analyses were conducted separately by ER status, since ER itself is strongly associated with pCR [23]. Multivariable analyses were adjusted by lymph node stage, tumour stage, histological grade, and HER2 status. Ordinal categories of node stage, tumour stage, and grade were modelled as continuous variables.

We addressed the possibility of selection bias due to missing data in multivariable models by deriving estimates based on multiply imputed datasets. Multiple imputation is a method for inferring the probable values of missing covariables repeatedly across several datasets, then deriving estimates for each dataset, and finally combining estimates in a manner that accounts for the variability between and within datasets. We imputed clinical covariables (tumour size or T stage, node status or N stage, grade, and HER2 status) across fifty datasets using the ice command in Stata [24]. Datasets were imputed separately for survival analyses and analyses of pCR. Quartiles of all immune cell subsets and clinical covariables were included in imputation models. An outcome variable was included for both survival imputation models (Nelson-Aalen cumulative hazard estimator and an outcome indicator variable) and pCR imputation models (pCR) [25]. Estimates were combined using Rubin’s rules.

We further addressed the possibility that variable selection based on univariable analysis may lead to exclusion of some variables that may be associated with outcome when confounding is properly controlled [26]. We accounted for the possibility of confounding by all immune cell subsets and clinical variables by fitting multivariable models (26 predictors) via penalised maximum likelihood using the package glmnet [27] with the penalisation factor selected based on cross-validation.

Associations between continuous and categorical variables were tested using the Kruskal-Wallis test. Correlations between immune cell subsets were evaluated using the Pearson correlation coefficient. For univariable analyses of the 22 immune cell subsets, adjustment for multiple testing was conducted by calculating *q*-values using the Benjamini-Hochberg method.

To investigate whether distinct classes of immune cell infiltration are present in different tumours and whether these classes are associated with different clinical outcome, we conducted hierarchical clustering of immune cell proportions. Values were rescaled to lie between zero (for the smallest value observed) and one (for the greatest value observed) for each cell type to ensure comparability between rare (low overall proportion) and abundant (high overall proportion) cell types. Hierarchical clustering of these data by Ward’s method was conducted across all samples. A combination of the Duda-Hart index and the associated pseudo-T-squared statistic was used to explore the likely number of distinct clusters in the data. The associations between clusters and clinical outcome were tested using the methods described above.

All analyses were conducted using R version 3.3 [28] or Stata SE version 14.1 (StataCorp). The complete dataset used for all analyses is available at https://github.com/cclab-brca.

## Results

### Performance of CIBERSORT across Studies

**Fig 2:**
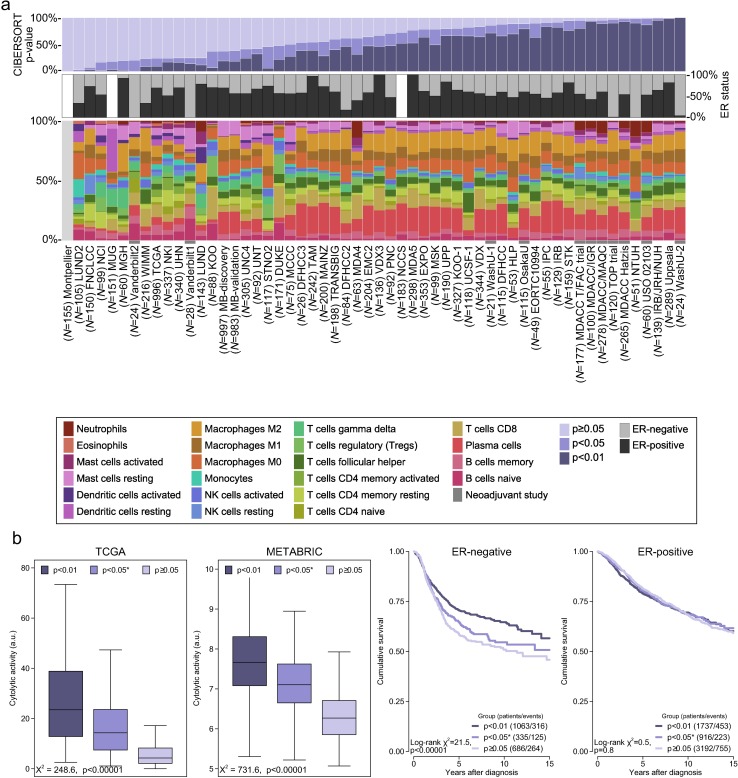
Summary of inferred immune cell subsets by study. (A) Bar charts summarising immune cell subset proportions against ER status and CIBERSORT *p*-value by study. (B) Box plots depicting the association between immune cytolytic activity and CIBERSORT *p*-value (outliers are not shown; depicted chi-squared statistics and *p*-values are from Kruskal-Wallis tests); survival plots of groups defined by CIBERSORT *p*-value separately by ER status (depicted chi-squared statistics and *p*-values are from log-rank tests). *0.01 ≤ *p* < 0.05. a.u., arbitrary units; ER, oestrogen receptor; NK cells, natural killer cells; TGCA, The Cancer Genome Atlas.

Fig 2A depicts a summary of the 56 included studies, totalling 10,988 cases of breast cancer. Gene expression was measured using a variety of platforms, as detailed in S1 Table. S1 Fig depicts the proportion of the 547 genes composing the signature matrix that were available for analysis by study. The mean number of genes represented was 447 (range 12–529). S2 and S3 Figs depict comparative summaries of CIBERSORT output across 100 cases from the METABRIC study where signature matrix genes were randomly deleted in increments of 10% (55 genes). S2 Fig shows that the empirical CIBERSORT *p*-value was highly sensitive to diminishing representation of the signature matrix, with over half of the samples (*n* = 53) with a CIBERSORT *p <* 0.05 at 87% representation but none at ≤27% representation. However, of the samples associated with *p <* 0.05, inferred cell proportions showed relatively small differences (S3 Fig), although this differed by cell type. These findings imply that, while variable representation of the signature matrix inevitably influences the accuracy of the inferred immune cell populations, this effect is not large when analyses are limited to cases with a CIBERSORT *p <* 0.05. At a threshold of CIBERSORT *p <* 0.05, 55% of samples (6,071/10,988) yielded data on infiltrating immune cells, while at a threshold of *p <* 0.01, 40% (4,385/10,988) of samples yielded data. Unless otherwise specified, analyses were restricted to samples with a CIBERSORT *p <* 0.05. Of these samples, the least variable cell type between studies was eosinophils (0%–3%), while the most variable was plasma cells (<1%–27%).

### CIBERSORT *p*-Values Reflect the Overall Proportion of Immune Cells

There were large differences in the proportions of samples at different *p*-value thresholds between studies, spanning the whole range of 0% through 100% of samples. This was only partly explained by ER status and representation of genes by different platforms. Notably, a larger proportion of samples from neoadjuvant studies were associated with a significant *p*-value. We hypothesized that the *p*-value derived by CIBERSORT, which tests the null hypothesis that none of the cells that comprise the signature matrix are present in a given sample [14], would reflect the proportion of a sample that comprises immune cells versus non-immune cells, where a greater proportion of non-immune cells would produce a correspondingly larger *p*-value. We tested this hypothesis against another in silico metric of inflammation (immune cytolytic activity [20]) in the two largest datasets: TCGA and METABRIC. Cytolytic activity has previously been defined by Rooney et al. as the geometric mean of *GZMA* and *PRF1* expression [20]. For TCGA we used cytolytic activity as computed by Rooney et al. For METABRIC we computed the geometric mean based on *GZMK* and *PRF1* expression because there was no probe for *GZMA*. However, *GZMA* and *GZMK* are highly correlated (Pearson correlation = 0.93 in the TCGA dataset). Cytolytic activity was most strongly correlated with the proportion of gamma-delta T cells (Pearson correlation = 0.51 in ER-positive and 0.49 in ER-negative disease) and M1 macrophages (Pearson correlation = 0.57 in ER-positive and 0.39 in ER-negative disease) in the TCGA and METABRIC studies at a CIBERSORT *p <* 0.05 (S4 and S5 Figs). Fig 2B depicts the strong ordinal relationship between different *p*-value thresholds and cytolytic activity in both the TCGA and METABRIC datasets. The relationship between cytolytic activity and the empirical CIBERSORT *p*-value strongly suggests that the *p*-value reflects the relative proportion of a sample composed of immune versus non-immune cells. We further tested this hypothesis by exploring the association between *p*-value thresholds and survival. Fig 2B shows that in ER-negative tumours, a *p <* 0.01, corresponding to a greater proportion of immune cells, was associated with significantly improved survival (log-rank *p <* 0.0001), while a *p ≥* 0.05 was associated with the poorest outcome, and *p* ≥ 0.01 but <0.05 showed an intermediate outcome, though not significantly different to that of the *p ≥* 0.05 group (log-rank *p* = 0.2). In contrast, in ER-positive disease, survival time did not differ significantly by the proportion of infiltrating immune cells as measured by CIBERSORT *p*-value (*p*_interaction_ < 0.001). This interaction between the prognostic effect of immune cells and ER status is well known [8,29,30], supporting the conclusion that CIBERSORT *p*-values reflect proportions of immune versus non-immune cells.

Proportions of different immune cells were weakly to moderately correlated. S6 and S7 Figs depict correlation matrices between immune cell subsets by ER status. Patterns of correlation were broadly similar irrespective of ER status. Among ER-positive tumours, neutrophils and natural killer cells showed the strongest positive correlation (Pearson correlation = 0.36), while resting memory T cells and CD8+ T cells showed the strongest negative correlation (Pearson correlation = −0.34). In ER-negative disease, T regulatory cells and CD8+ cells showed the strongest positive correlation (Pearson correlation = 0.33), and, as for ER-positive tumours, resting memory T cells and CD8+ T cells showed the strongest negative correlation (Pearson correlation = −0.33).

### Prognostic Subsets of Immune Cells

**Fig 3:**
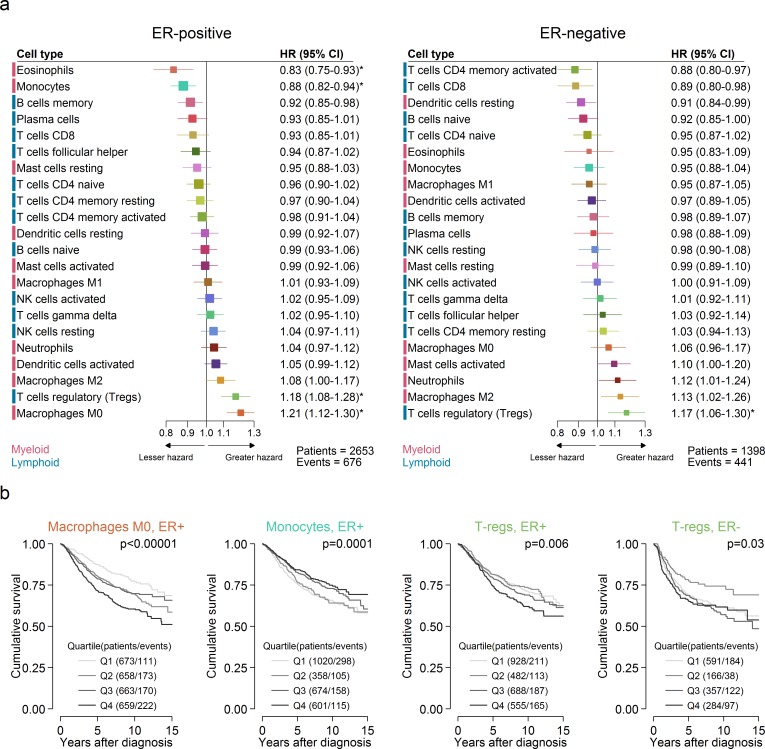
Prognostic associations of subsets of immune cells. (A) Unadjusted HRs (boxes) and 95% confidence intervals (horizontal lines) limited to cases with CIBERSORT *p*-value < 0.05. Box size is inversely proportional to the width of the confidence interval. Asterisks denote estimates with a *q*-value < 0.05. (B) Survival plots of quartiles of immune cell subsets. Depicted *p*-values are from log-rank tests. ER, oestrogen receptor; HR, hazard ratio; NK cells, natural killer cells.

Different immune cell subsets were associated with favourable outcome in ER-positive compared to ER-negative disease (Fig 3). Fig 3A depicts the unadjusted HRs and 95% confidence intervals for quartiles of cell type proportion by ER status. Restricting analyses to samples with a CIBERSORT *p <* 0.05, there were 2,653 patients with a median follow-up of 6.2 y for ER-positive disease, with the type of follow-up being relapse-free survival for 759 patients (171 events), distant metastasis-free survival for 976 patients (278 events), disease-specific survival for 529 patients (110 events), and overall survival for 389 patients (117 events). For ER-negative disease, there were 1,398 patients with a median follow-up of 4.4 y, with the type of follow-up being relapse-free survival for 298 patients (78 events), distant metastasis-free survival for 524 patients (180 events), disease-specific survival for 321 patients (92 events), and overall survival for 255 patients (91 events). Eosinophils (*p*_interaction_ by ER status = 0.052) and monocytes (*p*_interaction_ by ER status = 0.04) were significantly associated with improved outcome in ER-positive disease, whereas CD4+ activated memory T cells (*p*_interaction_ by ER status = 0.2) and CD8+ T cells (*p*_interaction_ by ER status = 0.03) were associated with improved outcome in ER-negative disease. In general, however, T cells tended to be associated with favourable outcome irrespective of ER status, though at varying significance. The exception to this general trend was T regulatory cells, which were associated with poorer outcome in both ER-positive (HR 1.18, 95% CI 1.08–1.28; *p <* 0.001) and ER-negative (HR 1.17, 95% CI 1.06–1.30; *p* = 0.002) disease, consistent with their proposed role as pro-tumourigenic immune suppressors [31,32]. Similarly, in ER-positive disease, both M0 and M2 macrophages were associated with poorer outcome, with a similar pattern in ER-negative disease, though non-significant for M0 macrophages (*p* = 0.2). Of these, M2 macrophages are thought to have an immune-suppressive pro-tumourogenic role [33,34]. Similar associations for M0 macrophages raise the possibility that there is some functional overlap between these two subsets of macrophages in the context of tumour-associated inflammation. Multivariable analyses adjusted for known prognostic factors revealed that in ER-positive disease (Table A in S1 Appendix), T regulatory cells (HR 1.15, 95% CI 1.04–1.26; *p* = 0.005), memory B cells (HR 0.92, 95% CI 0.84–1.00; *p* = 0.05), and M0 macrophages (HR 1.12, 95% CI 1.01–1.24; *p* = 0.04) all contributed to the model, with very similar imputed estimates (Table B in S1 Appendix). For comparison, Tables C and D in S1 Appendix show these same models for ER-negative disease. In ER-negative disease, M2 macrophages (HR 1.15, 95% CI 1.0–1.33; *p* = 0.05), CD8+ T cells (HR 0.87, 95% CI 0.75–1.00; *p* = 0.05), and T regulatory cells (HR 1.2, 95% CI 1.06–1.35; *p* = 0.005) all contributed to the adjusted model (Table E in S1 Appendix), with similar estimates based on multiple imputation, though CD8+ T cells ceased to be significant (HR 0.92, 95% CI 0.82–1.02; *p* = 0.1; Table F in S1 Appendix). For comparison, Tables G and H in S1 Appendix show these same models for ER-positive disease. Based on CIBERSORT data, Gentles al. previously reported neutrophils and plasma cells to be prognostic in both lung and breast cancer, with effects in opposing directions [35]. This observation was partly reproduced here insofar as the direction of effect was similar, with a greater proportion of neutrophils associated with poorer outcome in ER-negative disease and a greater proportion of plasma cells associated with better outcome in ER-positive disease (Fig 3). Tables I and J in S1 Appendix depict multivariable Cox regression models with penalised maximum likelihood estimation. Both models show attenuated but similar effects compared to the other multivariable models, with T regulatory cells associated with the largest point estimates in both ER-positive (HR = 1.13) and ER-negative disease (HR = 1.11).

### Variation in Prognostic Effect of Immune Cells by Molecular Subtype

We conducted exploratory subgroup analyses of the prognostic effect of all 22 immune cell subsets by molecular subtype defined by ER and HER2, and by the IntClust classifier based on genomic drivers [2] (S8 and S9 Figs). It should be noted that these two sets of subgroup analyses (based on ER/HER2 and IntClust, respectively) divide the same data in two different ways.

In subgroups defined by ER and HER2, significant heterogeneity of prognostic effect was observed for four cell subsets, of which three were of myeloid lineage. Activated memory T cells were associated with favourable outcome in all subgroups except ER+/HER2− tumours (HR 1.0, 95% CI 0.92–1.08), with the largest effect observed in the ER−/HER2+ subgroup (HR 0.73, 95% CI 0.60–0.89; *p*_heterogeneity_ = 0.022). In contrast, M0 macrophages were most strongly associated with poorer outcome in the ER+/HER2− subgroup (HR 1.25, 95% CI 1.15–1.37; *p*_heterogeneity_ = 0.024) compared to the other subgroups. Most strikingly, both activated dendritic cells and neutrophils showed the greatest heterogeneity of prognostic effect: a strong association with poor outcome in ER+/HER2+ tumours but not in the other subgroups. However, these cell types were not correlated in the ER+/HER2+ subgroup (Pearson correlation = 0.08) or overall (Pearson correlation = 0.07). Specifically, among this subset of patients, activated dendritic cells showed a strong association with poor outcome (HR 1.3, 95% CI 1.14–1.49), while in all other subgroups, including ER−/HER2+ tumours, the point estimate was less than one (*p*_heterogeneity_ = 0.0008). Similarly, neutrophils showed a clear association with poor outcome in the context of ER+/HER2+ disease (HR 1.37, 95% CI 1.18–1.60) but not in the other subgroups (*p*_heterogeneity_ = 0.0007).

Among subgroups defined by IntClust classification, plasma cells and activated mast cells showed significant heterogeneity of effect. Plasma cells were associated with favourable outcome among some ER-positive subgroups (IntClust 4+, IntClust 8, and IntClust 7) but not among other subgroups (*p*_heterogeneity_ = 0.03), while activated mast cells were associated with poorer outcome in IntClust 5 (HER2-positive disease), IntClust 8, and IntClust 9 but not others (*p*_heterogeneity_ = 0.03). These analyses warrant cautious interpretation as, despite a large overall sample size, they are relatively underpowered given the large number of subgroups.

### Tumours Lacking Immune Infiltration

**Fig 4:**
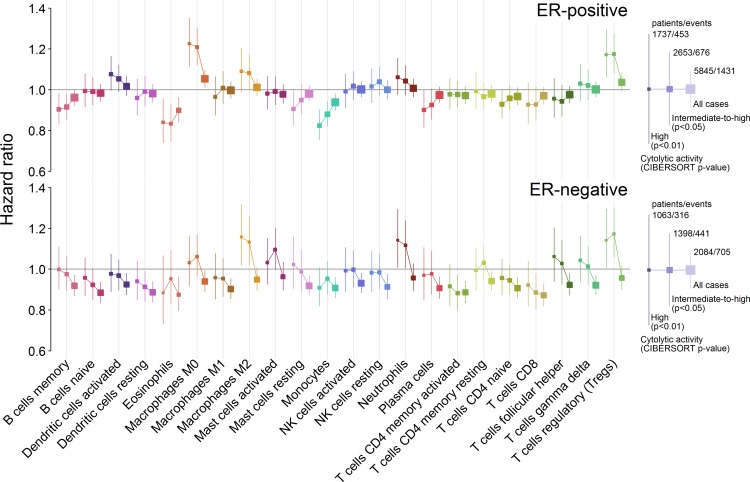
Hazard ratios for three overlapping populations defined by CIBERSORT *p*-value. Boxes represent hazard ratios, and vertical lines are 95% confidence intervals. Box size is inversely proportional to the width of the confidence interval. ER, oestrogen receptor; NK cells, natural killer cells.

**Fig 5:**
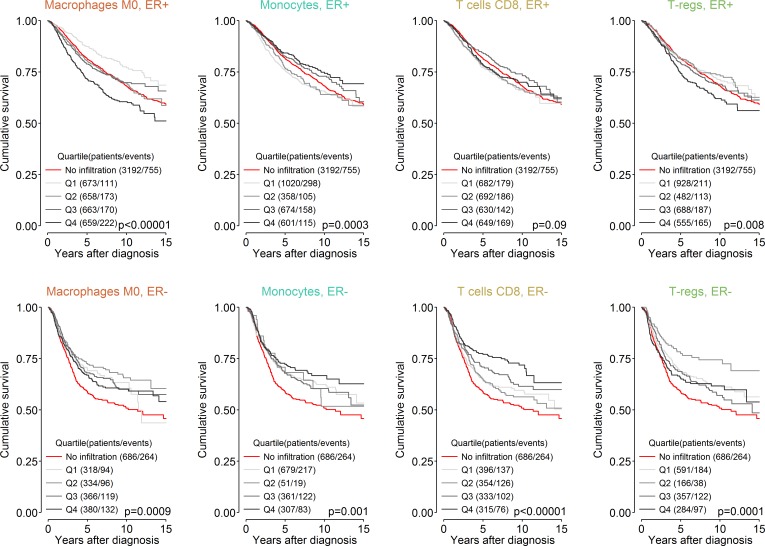
Survival plots highlighting the patient subgroup with tumours containing little or no immune infiltration by CIBERSORT *p*-value. Depicted *p*-values are from log-rank tests. ER, oestrogen receptor; T-regs, T regulatory cells.

Previous reports of the association between different immune cell types and outcome in breast cancer, including our large analysis of cytotoxic T cells [8], have generally found an association of immune cell types with survival in ER-negative breast cancer and little or no association in ER-positive breast cancer [8,36]. Our present findings partly contradicted these observations. Previous studies, however, included tumours lacking immune cells as well as those that contained them. Since our analyses were limited to tumours with at least moderate immune infiltration based on cytolytic activity (CIBERSORT *p <* 0.05), we hypothesized that comparison to a different reference population, i.e., one limited to tumours with moderate to high infiltration of immune cells, might explain why our observations differed from those previously reported. To test this hypothesis we undertook exploratory survival analyses where we investigated the association between all 22 cell subsets and outcome across three overlapping populations: those with a CIBERSORT *p <* 0.01, those with a CIBERSORT *p <* 0.05, and all cases irrespective of CIBERSORT *p*-value. For analyses of the whole population, cases with a *p ≥* 0.05 were taken as a separate reference group since they showed very low immune cell infiltration based on cytolytic activity. Fig 4 depicts the results of these analyses. In general, we found that in ER-negative disease, as the size of the population increased to include tumours with low or no infiltration, the prognostic effect of the majority of immune cell types shifted toward improved outcome, whereas in ER-positive disease, the shift was toward the null. This was observed irrespective of whether the point estimate for the high infiltration (CIBERSORT *p <* 0.01) population was more or less than one to begin with: it fell closer to one in ER-positive disease and fell below one in ER-negative disease. This finding indicates a general trend that differs by ER status. In ER-positive disease, tumours lacking immune infiltration were associated with an outcome that was intermediate or similar to tumours with low or high infiltration, whereas in ER-negative disease, tumours lacking immune infiltration were associated with a poorer outcome compared to tumours with infiltration, irrespective of its degree. This is further illustrated in Fig 5, which depicts survival plots highlighting the population with tumours showing low to no infiltration. We speculate that this finding may explain many of the contradictory reports of the prognostic effect of different immune cell subsets in breast cancer, and possibly in other solid tumours [37]. Moreover, it potentially has profound implications for inclusion of immune infiltrates as part of clinical prognostic models.

### Immune Predictors of Response to Chemotherapy

**Fig 6:**
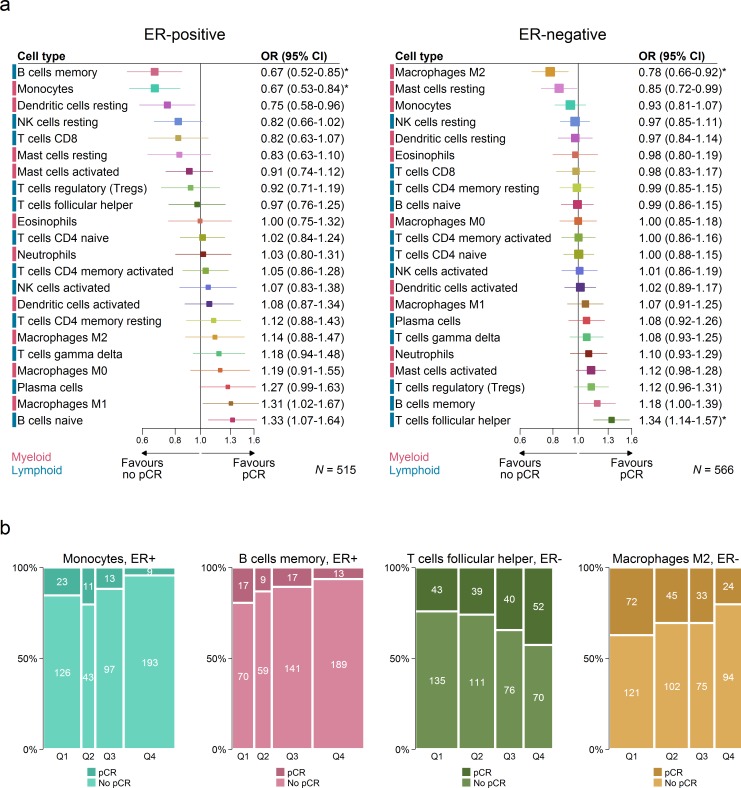
Association between immune cell subsets and response to neoadjuvant chemotherapy. (A) Boxes represent ORs from unadjusted logistic regression models. Horizontal lines are 95% confidence intervals. Box size is inversely proportional to the width of the confidence interval. Asterisks denote estimates with *q*-value < 0.05. (B) Spine plots depicting the association between quartiles of immune cell subsets and pCR. ER, oestrogen receptor; NK cells, natural killer cells; OR, odds ratio; pCR, pathological complete response.

We used data from studies of neoadjuvant chemotherapy to assess the association between subsets of immune cells and pCR to chemotherapy (Fig 6). pCR is associated with improved survival, and this association is greatest in aggressive subtypes of breast cancer, which are more often ER negative [23]. Therefore we conducted analyses separately by ER status. In ER-negative tumours, T follicular helper cells (Tfh cells) showed the strongest association with pCR (odds ratio [OR] 1.34, 95% CI 1.14–1.57; *p <* 0.001; *p*_interaction_ by ER status = 0.03), while M2 macrophages showed the strongest association with a lack of pCR and, therefore, resistance to chemotherapy (OR 0.78, 95% CI 0.66–0.92; *p* = 0.003; *p*_interaction_ by ER status = 0.02). This may partly explain the association between M2 macrophages and poorer outcome in ER-negative disease. Multivariate analysis in ER-negative disease (Table O in S1 Appendix) revealed that both M2 macrophages (OR 0.75, 95% CI 0.58–0.96; *p* = 0.02) and resting mast cells (OR 0.77, 95% CI 0.61–0.97; *p* = 0.03) contributed to the adjusted model in the complete case analysis (*n* = 332), but estimates based on multiple imputation (Table P in S1 Appendix) revealed that M2 macrophages (OR 0.83, 95% CI 0.96–1.00; *p* = 0.05), memory B cells (OR 1.2, 95% CI 1.01–1.42; *p* = 0.04), and Tfh cells (OR 1.31, 95% CI 1.11–1.56; *p* = 0.002) all contributed to the adjusted model (*n* = 566). In ER-positive disease, both memory B cells and monocytes showed a strong association with a lack of pCR. However, in survival analyses, both these cell types were associated with better rather than worse outcome. The basis of this apparent contradiction in ER-positive breast cancer is not known, and pCR has not been generally accepted as an endpoint for assessing the effectiveness of chemotherapy in ER-positive disease [23]. Estimates based on multivariable models with penalised maximum likelihood estimation did not show any associations between the 26 predictors and pCR. This is likely to be related, in part, to the modest power of these models relative to the number of predictors.

### Immune Clusters Associated with Outcome and Patient Age

**Fig 7:**
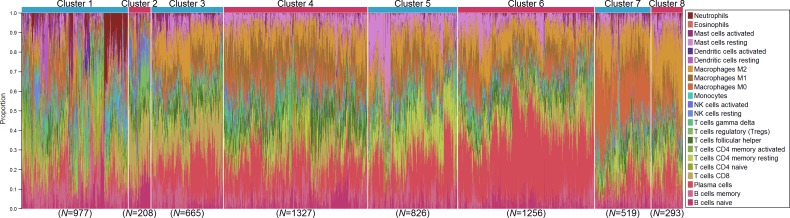
Hierarchical clustering of all samples based on immune cell proportions. Stacked bar charts of samples ordered by cluster assignment. NK cells, natural killer cells.

**Fig 8:**
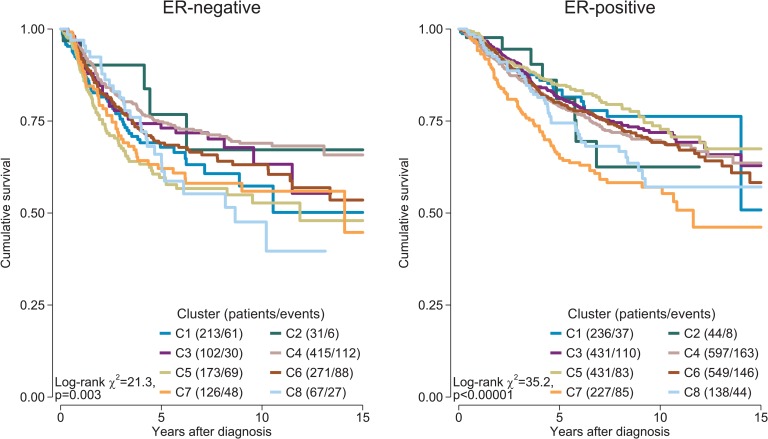
Survival plots by cluster separately for ER-positive and ER-negative disease. Depicted *p*-values are from log-rank tests. ER, oestrogen receptor.

We investigated whether distinct patterns of immune infiltration could be discerned based on the 22 immune cell proportions by conducting hierarchical clustering of all samples. We interpreted the Duda-Hart index and the associated pseudo-T-squared statistic to indicate eight clusters in the data (S10 Fig). Cell proportions by cluster are depicted in Fig 7, and their distributions as box plots in S11 and S12 Figs. Clusters were associated with distinct patterns of survival in ER-positive and ER-negative disease (Fig 8). Cluster 7, defined by high levels of M0 and M2 macrophages, and cluster 8, defined by a high level of M2 macrophages though relatively low level of M0 and M1 macrophages, were both associated with poor outcome in ER-negative and ER-positive disease. In contrast, cluster 5, defined by moderate levels of M0 and M1 macrophages and plasma cells and a high level of resting memory T cells, was associated with poor outcome in ER-negative tumours but with the best outcome in ER-positive disease. Clusters were significantly, but modestly, associated with ER status (*p <* 0.001) and with IntClust subtype (*p <* 0.001; S13 Fig). The association with ER status was largely due to clusters 3 and 4. Cluster 3 was particularly enriched for ER-positive tumours (77% compared to 62% overall), while cluster 4 was enriched for ER-negative tumours (45% compared to 38% overall). Immune clusters were also associated with patient age in ER-positive disease (*p <* 0.001), with a weak association in ER-negative disease (*p* = 0.05). Women with ER-positive tumours assigned to cluster 1 (defined by high levels of neutrophils and CD8+ T cells) were on average 53 y old at diagnosis, while those with tumours assigned to cluster 3 (defined by high levels of M2 macrophages, CD8+ T cells, and plasma cells) were on average 60 y old. Collectively, these findings suggest that there is considerable variability in the nature of the immune infiltrate across breast tumours—partly determined by molecular characteristics of the primary tumour—and that this influences clinical outcome.

## Discussion

Based on deconvolution of bulk gene expression data from nearly 11,000 cases of breast cancer, we uncovered distinct patterns of immune infiltration between tumours, complex associations with clinical outcome for different subsets of immune cells that depend on ER status, and immune mediators of both sensitivity and resistance to chemotherapy.

Advances in computational methods have reinvigorated the potential of large public repositories of genomic data collected over the past two decades. Using a state-of-the-art deconvolution algorithm, CIBERSORT [14], to infer the proportions of 22 immune cell subsets from tumour transcriptomes, we have conducted, to our knowledge, the most comprehensive analysis of the clinical impact of the immune response in breast cancer to date. We found that some immune suppressor cells (T regulatory cells and M2 macrophages) were associated with poorer outcome but that M0 macrophages too showed similar associations with survival. Previous experiments have shown that tumours may attract T regulatory cells to evade the immune response [31,32], and although subsets of T regulatory cells with different functions in cancer continue to be discovered [38], our findings support the tumour-promoting role of T regulatory cells. The distinction between “polarised states” of macrophages is, however, the subject of some dispute [34]. The M1 (activated; anti-tumoural) and M2 (alternatively activated; pro-tumoural) phenotypes are associated with distinct immunoregulatory functions, but it has been argued that they represent extremes of a spectrum of functional states rather than truly distinct cell types [34]. Tumours are likely to contain macrophages in a variety of such states, and our finding of an association between M0 macrophages and poorer outcome may reflect this gradation of function. We also found that the proportions of M0, M1, and M2 macrophages defined several immune cell signatures in our clustering analysis, with prognostic implications. Moreover, higher proportions of M2 macrophages were associated with a lack of response to chemotherapy in ER-negative tumours, suggesting that they may mediate resistance mechanisms—a conclusion supported by previous preclinical experiments [39]. Collectively, these findings demonstrate the potential of tumour-associated macrophages as biomarkers in breast cancer. In particular, they highlight the necessity of accounting for the diversity of macrophages’ functional states rather than treating them all equally. This is of special importance because treatments to combat the tumour-promoting roles of macrophages are already in early phase clinical trials [40]. These approaches block colony stimulating factor 1 (CSF1) signalling by targeting colony stimulating factor 1 receptor (CSF1R) [39,41]. Preclinical evidence also supports the potential of CSF1R inhibition in breast cancer in conjunction with conventional chemotherapy [39]. Inhibition of CSF1R has been shown not to kill macrophages but to lead to a change in their functional state to a state thought to be anti-tumoural [34,42]. In this respect, this approach might be considered a differentiation therapy of the tumour microenvironment, again emphasising the need for practical biomarkers that account for the variety of functional states of macrophages in cancer tissue.

We also found Tfh cells to be strongly associated with response to chemotherapy in ER-negative breast cancer. Tfh cells play an important role in the maintenance and development of germinal centres and the selective evolution of high-affinity plasma cells and memory B cells from antigen-specific B cells. They have not been widely investigated in solid tumours, but, based on genomic and histological analyses, they have recently been shown to play a role in breast tumours with extensive immune infiltration and, in particular, in the response to chemotherapy [43]. Whether this effect is exerted through tumour-specific antibody-dependent mechanisms is uncertain, but there is some evidence for tumour-antigen-specific responses by B cells in breast cancer [44,45], as well as observations of an association between plasma cells and outcome across solid tumours [35,46,47]. Moreover, the fact that cross-reactive antibodies targeting tumour neoepitopes underpin some paraneoplastic syndromes such as scleroderma [48] strongly implies that tumour-antigen-specific antibody production can be part of the host response to tumours. The presence of Tfh cells in breast cancer was also shown to be a reliable indicator of an organised immune response including tertiary lymphoid structures [43]. Our findings further support this observation since memory B cells were also associated with response to chemotherapy in ER-negative tumours (Fig 6), with the strength of the association second only to that of Tfh cells. In the clinical setting, these findings lend support to the use of tumour-infiltrating lymphocytes as predictors of response to neoadjuvant chemotherapy but also suggest that specifically accounting for the abundance of Tfh cells in dense immune infiltrates may substantially improve predictive accuracy.

We found that tumours with little or no immune infiltration showed fundamental differences in patterns of patient survival based on ER status. In ER-negative disease, these tumours were associated with poorer outcome compared to tumours with immune infiltration regardless of immune cell subset. Poorer outcome in patients with tumours that have failed to evince an immune infiltrate is consistent with the idea that, in general, the immune response is tumouricidal, albeit variably effective depending on its context and composition. For example, increasing proportions of M0 macrophages were associated with poorer outcome, but the group lacking immune infiltration was associated with survival poorer than that of patients with tumours in the highest quartile of M0 macrophage proportion, rather than better survival than the group in the first quartile, which is what might be expected if the association of survival with M0 macrophages followed a strictly ordinal pattern. Whether these ER-negative tumours lacking an immune infiltrate have been extensively immunoedited [49] and are therefore no longer immunogenic, whether they enact an alternative approach to evade immune infiltration, or whether the host is not able to mount an immune response is unknown. However, their natural history appears consistent with the view of the immune response as an extrinsic tumour suppressor, in contrast to the findings in the group of ER-positive tumours that lack immune infiltration. This group was not associated with a survival pattern easily explained by the immune response. Women with ER-positive tumours lacking immune infiltration had a survival outcome intermediate between that of women with tumours with high and low infiltration, irrespective of cell type. This observation is more suggestive of tumours that do not evince an immune response from conception rather than of immunoedited tumours. It also implies that tumours lacking an immune response are not next in sequence to those with a minimal response but differ more fundamentally, and should be excluded from relevant biomarker analyses. Finally, this group of patients is likely to gain the least from new immune checkpoint inhibitors unless a way of inducing an effective immune response is coupled with this approach.

Previous immune profiling studies in breast cancer have used either genomic data or in situ histological analyses to quantify the immune response [8,29,36,50,51]. These approaches have confirmed the prognostic significance of the immune response, particularly in ER-negative disease, but have been of limited resolution, often including only one or two cell types. While this has enabled very large scale analyses and candidate clinical assay development, much of the functional variation implicit in the immune response, and its clinical importance, is missed. Studies using multiparametric flow cytometry and immunofluorescence have produced an expanded, but still limited, view of the immune response at cellular resolution [52], but have been restricted to very small sample sizes, precluding analysis of association with clinical outcome. One recent analysis of genomic data also deconvolved tumour transcriptomes to estimate the presence of 230 murine haematopoietic lineage profiles, generating “cell lineage scores” for patients, and investigated their association with outcome [53]. While an important advance on previous work, this approach has not been as extensively validated as CIBERSORT, and the analysis comprised a much smaller sample size than the present work and did not directly address associations with treatment response. Recent pan-cancer analyses using quantitative genomic approaches have also uncovered important associations between immune cell subsets and survival across tumour types [35,54]. Our study further advances knowledge of the clinical impact of the immune response, particularly highlighting diverse associations with outcome related to different functional cell subsets and, importantly, accounts for the significance of molecular subtype.

This was a large analysis of tumour samples from a great breadth of studies in which we enumerated the immune response in detail. Our conclusions are likely, therefore, to be reliable and generalizable. This is the main strength of the study; however, in increasing our sample size we made use of historic studies, some of which were profiled using platforms not formally validated for use with CIBERSORT, which is the main limitation of this work. Therefore, the reliability of the inferred immune infiltrate in some studies is uncertain. Similarly, in our effort to derive precise and reliable estimates of association with clinical outcome, we collated diverse and heterogeneous studies with different types of follow-up, which is a second limitation. While this is not ideal, it should be noted that relapse or metastasis is highly correlated with disease-specific survival, and our conclusions are substantially more reliable because our approach increased statistical power. A large proportion of patients who experience disease relapse or metastasis will ultimately succumb to their disease, though at a later time point, which, all other factors being equal, may attribute slightly greater weight to relapse-free or metastasis-free survival. It is likely that this effect will be larger for ER-positive disease because patients with metastatic ER-positive breast cancer tend to experience longer survival than those with ER-negative breast cancer. Another related limitation is the relatively short average follow-up for each patient: the median follow-up was 6.2 y for ER-positive patients and 4.4 y for ER-negative patients. For both subsets this falls short of capturing all disease-related events. Patients with ER-positive disease continue to experience relapse well beyond 10 y, while those with ER-negative disease invariably experience relapse within the first 5 y [21], and some of these events were not represented in this analysis. While the diversity of studies included in the analysis increases the generalizability our findings, it is also a limitation insofar as patients are likely to have experienced differences in clinical care owing to differences in date of diagnosis, clinical setting, geography, and treatment. It is, however, unlikely that these differences affected the inferred immune cell populations of primary tumours; if anything, these differences are likely to diminish associations between the immune response and clinical outcome, raising the possibility that our observations underestimate these effects and may include false negatives. Finally, the associations reported here arise from discovery analyses and require validation in independent studies.

Our analysis of 22 immune cell subsets in breast cancer has revealed important associations with clinical outcome that have the potential to identify patients who could benefit from immunotherapies, as well as highlighting possible targets for new drugs. Coupling reliable deconvolution algorithms with large-scale genomic data has the potential to further uncover the clinical and biological significance of the plethora of non-cancer cells that comprise the tumour microenvironment in breast cancer.

## Supporting Information

S1 Appendix Multivariable regression models. (DOCX)

S1 Fig Bar chart of the proportion of the 547 signature matrix genes available by study. (PDF)

S2 Fig Box plot of the distribution of CIBERSORT *p*-value and average Pearson correlation for nine runs using datasets with progressively fewer (10% increments) signature matrix genes for 100 cases from the METABRIC study. Outliers not shown.
(PDF)

S3 Fig Distribution of 22 immune cell subsets for 100 cases from the METABRIC study with decreasing representation of signature matrix genes. Outliers not shown.
(PDF)

S4 Fig Correlation matrix of all 22 immune cell proportions and immune cytolytic activity in the TCGA and METABRIC datasets for ER-positive disease. Variables have been ordered by average linkage clustering. For comparison, cytolytic activity has been rescaled to range between zero and one separately in each study.
(PDF)

S5 Fig Correlation matrix of all 22 immune cell proportions and immune cytolytic activity in the TCGA and METABRIC datasets for ER-negative disease. Variables have been ordered by average linkage clustering. For comparison, cytolytic activity has been rescaled to range between zero and one separately in each study.
(PDF)

S6 Fig Correlation matrix of all 22 immune cell proportions in ER-positive disease. Variables have been ordered by average linkage clustering.
(PDF)

S7 Fig Correlation matrix of all 22 immune cell proportions in ER-positive disease. Variables have been ordered by average linkage clustering.
(PDF)

S8 Fig Subgroup survival analyses by ER and HER2 status. (PDF)

S9 Fig Subgroup survival analyses by IntClust classification. (PDF)

S10 Fig Line plot of Duda-Hart index and associated pseudo-T-squared statistic. (PDF)

S11 Fig Box plots depicting the distribution of cell type proportion by immune cluster. Proportions, by each cell type, have been rescaled to range between zero and one.
(PDF)

S12 Fig Box plots depicting the distribution of each immune cell type across eight immune clusters. Proportions have been rescaled to range between zero and one. Outliers not shown.
(PDF)

S13 Fig Associations between immune cell cluster, molecular subtype, and patient age. (Top panel) Spine plots of the relationship between immune cluster and IntClust subtype. (Bottom panel) Box plots of the distribution of age at diagnosis by immune cluster. Outliers are not shown. *p*-Values are from Kruskal-Wallis tests.
(PDF)

S1 Table Summary of included studies. (XLSX)

S2 Table Distributions of clinical factors and molecular subtypes by study. (XLSX)

S3 Table STROBE checklist. (DOC)

## Abbreviations

ER: oestrogen receptor
HR: hazard ratio
OR: odds ratio
pCR: pathological complete response
Tfh cell: T follicular helper cell
TGCA: The Cancer Genome Atlas
