# Supplementary material for: Patterns of Immune Infiltration in Breast Cancer and Their Clinical Implications: A Gene-Expression-Based Retrospective Study
Source: PLoS Med. 2016 Dec 13;13(12):e1002194. doi: 10.1371/journal.pmed.1002194 (PMC5154505; doi:10.1371/journal.pmed.1002194)
Supplement: S1 Appendix — (DOCX) [file pmed.1002194.s001.docx]

**Supplementary Table A: Multivariable Cox regression survival analysis adjusted for clinical factors in ER-positive disease (complete case analysis). Selected immune cell subsets are those significantly associated with outcome in univariable analysis of ER-positive breast cancer.**

| **ER-positive (CCA)** | | | | |
| --- | --- | --- | --- | --- |
| **Variable** | **Categories** | **Hazard ratio** | **95% CI** | **P-value** |
| Eosinophils | Quartiles | 0.94 | 0.83-1.07 | 0.35 |
| Monocytes | Quartiles | 0.97 | 0.89-1.06 | 0.54 |
| B cells memory | Quartiles | 0.92 | 0.84-1.00 | 0.05 |
| T cells regulatory (Tregs) | Quartiles | 1.15 | 1.04-1.26 | 0.005 |
| Macrophages M0 | Quartiles | 1.12 | 1.01-1.24 | 0.04 |
| Node status | Negative, Positive | 1.88 | 1.49-2.37 | <0.00001 |
| Tumour size | <10mm, 10-19mm, 20-29mm, 30-49mm, 50+mm | 1.34 | 1.19-1.50 | <0.00001 |
| Grade | 1,2,3 | 1.4 | 1.19-1.64 | 0.00004 |
| HER2 status | Negative, Positive | 1.45 | 1.14-1.85 | 0.003 |
| **Observations** | 1763 |  |  |  |
| **Subjects** | 451 |  |  |  |

**Supplementary Table B: Multivariable Cox regression survival analysis adjusted for clinical factors in ER-positive disease (multiple imputation). Selected immune cell subsets are those significantly associated with outcome in univariable analysis of ER-positive breast cancer.**

| **ER-positive (Imputed M=50)** | | | | |
| --- | --- | --- | --- | --- |
| **Variable** | **Categories** | **Hazard ratio** | **95% CI** | **P-value** |
| Eosinophils | Quartiles | 0.91 | 0.81-1.02 | 0.11 |
| Monocytes | Quartiles | 0.99 | 0.91-1.06 | 0.7 |
| B cells memory | Quartiles | 0.94 | 0.87-1.00 | 0.07 |
| T cells regulatory (Tregs) | Quartiles | 1.11 | 1.03-1.21 | 0.009 |
| Macrophages M0 | Quartiles | 1.13 | 1.04-1.23 | 0.005 |
| Node status | Negative, Positive | 1.84 | 1.50-2.26 | <0.00001 |
| Tumour size | <10mm, 10-19mm, 20-29mm, 30-49mm, 50+mm | 1.31 | 1.17-1.47 | <0.00001 |
| Grade | 1,2,3 | 1.36 | 1.17-1.58 | 0.00005 |
| HER2 status | Negative, Positive | 1.4 | 1.14-1.73 | 0.001 |
| **Observations** | 2653 |  |  |  |

**Supplementary Table C: Multivariable Cox regression survival analysis adjusted for clinical factors in ER-negative disease (complete case analysis). Selected immune cell subsets are those significantly associated with outcome in univariable analysis of ER-positive breast cancer.**

| **ER-negative (CCA)** | | | | |
| --- | --- | --- | --- | --- |
| **Variable** | **Categories** | **Hazard ratio** | **95% CI** | **P-value** |
| Eosinophils | Quartiles | 0.95 | 0.77-1.18 | 0.67 |
| Monocytes | Quartiles | 0.92 | 0.82-1.04 | 0.17 |
| B cells memory | Quartiles | 1.08 | 0.95-1.22 | 0.24 |
| T cells regulatory (Tregs) | Quartiles | 1.19 | 1.05-1.34 | 0.008 |
| Macrophages M0 | Quartiles | 1.07 | 0.93-1.23 | 0.34 |
| Node status | Negative, Positive | 2.21 | 1.60-3.05 | <0.00001 |
| Tumour size | <10mm, 10-19mm, 20-29mm, 30-49mm, 50+mm | 1.32 | 1.13-1.53 | 0.0004 |
| Grade | 1,2,3 | 1.2 | 0.90-1.62 | 0.22 |
| HER2 status | Negative, Positive | 1.04 | 0.78-1.40 | 0.77 |
| **Observations** | 755 |  |  |  |
| **Subjects** | 240 |  |  |  |

**Supplementary Table D: Multivariable Cox regression survival analysis adjusted for clinical factors in ER-negative disease (multiple imputation). Selected immune cell subsets are those significantly associated with outcome in univariable analysis of ER-positive breast cancer.**

| **ER-negative (Imputed M=50)** | | | | |
| --- | --- | --- | --- | --- |
| **Variable** | **Categories** | **Hazard ratio** | **95% CI** | **P-value** |
| Eosinophils | Quartiles | 0.99 | 0.86-1.14 | 0.91 |
| Monocytes | Quartiles | 0.96 | 0.88-1.05 | 0.37 |
| B cells memory | Quartiles | 0.98 | 0.89-1.07 | 0.66 |
| T cells regulatory (Tregs) | Quartiles | 1.16 | 1.05-1.29 | 0.003 |
| Macrophages M0 | Quartiles | 1.01 | 0.91-1.12 | 0.8 |
| Node status | Negative, Positive | 1.81 | 1.38-2.38 | 0.00002 |
| Tumour size | <10mm, 10-19mm, 20-29mm, 30-49mm, 50+mm | 1.21 | 1.06-1.39 | 0.006 |
| Grade | 1,2,3 | 1.29 | 1.01-1.65 | 0.04 |
| HER2 status | Negative, Positive | 1.03 | 0.81-1.32 | 0.79 |
| **Observations** | 1398 |  |  |  |

**Supplementary Table E: Multivariable Cox regression survival analysis adjusted for clinical factors in ER-negative disease (complete case analysis). Selected immune cell subsets are those significantly associated with outcome in univariable analysis of ER-negative breast cancer.**

| **ER-negative (CCA)** | | | | |
| --- | --- | --- | --- | --- |
| **Variable** | **Categories** | **Hazard ratio** | **95% CI** | **P-value** |
| T cells CD4 memory activated | Quartiles | 0.98 | 0.85-1.14 | 0.83 |
| T cells CD8 | Quartiles | 0.87 | 0.75-1.00 | 0.05 |
| Dendritic cells resting | Quartiles | 0.93 | 0.83-1.04 | 0.21 |
| Neutrophils | Quartiles | 1 | 0.86-1.15 | 0.97 |
| Macrophages M2 | Quartiles | 1.15 | 1.00-1.33 | 0.05 |
| T cells regulatory (Tregs) | Quartiles | 1.2 | 1.06-1.35 | 0.005 |
| Node status | Negative, Positive | 2.2 | 1.59-3.03 | <0.00001 |
| Tumour size | <10mm, 10-19mm, 20-29mm, 30-49mm, 50+mm | 1.3 | 1.12-1.51 | 0.0007 |
| Grade | 1,2,3 | 1.19 | 0.88-1.61 | 0.25 |
| HER2 status | Negative, Positive | 1.04 | 0.77-1.40 | 0.81 |
| **Observations** | 755 |  |  |  |
| **Subjects** | 240 |  |  |  |

**Supplementary Table F: Multivariable Cox regression survival analysis adjusted for clinical factors in ER-negative disease (multiple imputation). Selected immune cell subsets are those significantly associated with outcome in univariable analysis of ER-negative breast cancer.**

| **ER-negative (Imputed M=50)** | | | | |
| --- | --- | --- | --- | --- |
| **Variable** | **Categories** | **Hazard ratio** | **95% CI** | **P-value** |
| T cells CD4 memory activated | Quartiles | 0.94 | 0.85-1.04 | 0.22 |
| T cells CD8 | Quartiles | 0.92 | 0.82-1.02 | 0.11 |
| Dendritic cells resting | Quartiles | 0.95 | 0.87-1.03 | 0.24 |
| Neutrophils | Quartiles | 1.04 | 0.94-1.15 | 0.42 |
| Macrophages M2 | Quartiles | 1.15 | 1.03-1.27 | 0.01 |
| T cells regulatory (Tregs) | Quartiles | 1.16 | 1.05-1.28 | 0.004 |
| Node status | Negative, Positive | 1.79 | 1.36-2.36 | 0.00005 |
| Tumour size | <10mm, 10-19mm, 20-29mm, 30-49mm, 50+mm | 1.21 | 1.06-1.39 | 0.006 |
| Grade | 1,2,3 | 1.29 | 1.01-1.66 | 0.04 |
| HER2 status | Negative, Positive | 1.05 | 0.82-1.34 | 0.7 |
| **Observations** | 1398 |  |  |  |

**Supplementary Table G: Multivariable Cox regression survival analysis adjusted for clinical factors in ER-positive disease (complete case analysis). Selected immune cell subsets are those significantly associated with outcome in univariable analysis of ER-negative breast cancer.**

| **ER-positive (CCA)** | | | | |
| --- | --- | --- | --- | --- |
| **Variable** | **Categories** | **Hazard ratio** | **95% CI** | **P-value** |
| T cells CD4 memory activated | Quartiles | 1 | 0.91-1.09 | 0.92 |
| T cells CD8 | Quartiles | 0.89 | 0.79-1.00 | 0.05 |
| Dendritic cells resting | Quartiles | 1.02 | 0.93-1.12 | 0.68 |
| Neutrophils | Quartiles | 0.93 | 0.85-1.02 | 0.12 |
| Macrophages M2 | Quartiles | 1.15 | 1.04-1.28 | 0.007 |
| T cells regulatory (Tregs) | Quartiles | 1.17 | 1.07-1.29 | 0.0008 |
| Node status | Negative, Positive | 1.91 | 1.51-2.40 | <0.00001 |
| Tumour size | <10mm, 10-19mm, 20-29mm, 30-49mm, 50+mm | 1.33 | 1.19-1.50 | <0.00001 |
| Grade | 1,2,3 | 1.49 | 1.27-1.75 | <0.00001 |
| HER2 status | Negative, Positive | 1.48 | 1.16-1.90 | 0.002 |
| **Observations** | 1763 |  |  |  |
| **Subjects** | 451 |  |  |  |

**Supplementary Table H: Multivariable Cox regression survival analysis adjusted for clinical factors in ER-positive disease (multiple imputation). Selected immune cell subsets are those significantly associated with outcome in univariable analysis of ER-negative breast cancer.**

| **ER-positive (Imputed M=50)** | | | | |
| --- | --- | --- | --- | --- |
| **Variable** | **Categories** | **Hazard ratio** | **95% CI** | **P-value** |
| T cells CD4 memory activated | Quartiles | 0.96 | 0.89-1.03 | 0.21 |
| T cells CD8 | Quartiles | 0.93 | 0.85-1.03 | 0.16 |
| Dendritic cells resting | Quartiles | 1.04 | 0.96-1.12 | 0.38 |
| Neutrophils | Quartiles | 1 | 0.93-1.07 | 0.95 |
| Macrophages M2 | Quartiles | 1.13 | 1.04-1.23 | 0.005 |
| T cells regulatory (Tregs) | Quartiles | 1.14 | 1.05-1.23 | 0.002 |
| Node status | Negative, Positive | 1.88 | 1.53-2.31 | <0.00001 |
| Tumour size | <10mm, 10-19mm, 20-29mm, 30-49mm, 50+mm | 1.32 | 1.18-1.47 | <0.00001 |
| Grade | 1,2,3 | 1.45 | 1.25-1.67 | <0.00001 |
| HER2 status | Negative, Positive | 1.47 | 1.19-1.81 | 0.0004 |
| **Observations** | 2653 |  |  |  |

**Supplementary Table I: Multivariable Cox-regression model based on penalised maximum likelihood estimation (complete case analysis) in ER-positive disease.**

| **ER-positive** | | |
| --- | --- | --- |
| **Variable** | **Categories** | **Hazard ratio*** |
| B cells naive | Quartiles | 1.04 |
| B cells memory | Quartiles | 0.97 |
| Plasma cells | Quartiles | 0.93 |
| T cells CD8 | Quartiles | - |
| T cells CD4 naive | Quartiles | - |
| T cells CD4 memory resting | Quartiles | 0.98 |
| T cells CD4 memory activated | Quartiles | - |
| T cells follicular helper | Quartiles | 0.97 |
| T cells regulatory (Tregs) | Quartiles | 1.13 |
| T cells gamma delta | Quartiles | - |
| NK cells resting | Quartiles | 1.04 |
| NK cells activated | Quartiles | - |
| Monocytes | Quartiles | 0.97 |
| Macrophages M0 | Quartiles | 1.09 |
| Macrophages M1 | Quartiles | - |
| Macrophages M2 | Quartiles | 1.06 |
| Dendritic cells resting | Quartiles | 1.08 |
| Dendritic cells activated | Quartiles | - |
| Mast cells resting | Quartiles | 0.97 |
| Mast cells activated | Quartiles | - |
| Eosinophils | Quartiles | - |
| Neutrophils | Quartiles | - |
| Node status | Negative, Positive | 1.46 |
| Tumour size | <10mm, 10-19mm, 20-29mm, 30-49mm, 50+mm | 1.3 |
| Grade | 1,2,3 | 1.41 |
| HER2 status | Negative, Positive | 1.3 |
| Observations | 1763 |  |
| Events | 457 |  |

*A statistical significance test has not yet been described for this method, however non-zero values indicate variables with an important contribution to the multi-variable model. Variables with missing estimates do not contribute to the model and correspond to coefficients of zero (hazard ratio of 1).

**Supplementary Table J: Multivariable Cox-regression model based on penalised maximum likelihood estimation (complete case analysis) in ER-negative disease.**

| **ER-negative** | | |
| --- | --- | --- |
| **Variable** | **Categories** | **Hazard ratio*** |
| B cells naive | Quartiles | 0.94 |
| B cells memory | Quartiles | 1.04 |
| Plasma cells | Quartiles | - |
| T cells CD8 | Quartiles | 0.95 |
| T cells CD4 naive | Quartiles | - |
| T cells CD4 memory resting | Quartiles | - |
| T cells CD4 memory activated | Quartiles | - |
| T cells follicular helper | Quartiles | 0.91 |
| T cells regulatory (Tregs) | Quartiles | 1.11 |
| T cells gamma delta | Quartiles | - |
| NK cells resting | Quartiles | - |
| NK cells activated | Quartiles | - |
| Monocytes | Quartiles | 0.94 |
| Macrophages M0 | Quartiles | - |
| Macrophages M1 | Quartiles | - |
| Macrophages M2 | Quartiles | 1.06 |
| Dendritic cells resting | Quartiles | - |
| Dendritic cells activated | Quartiles | - |
| Mast cells resting | Quartiles | - |
| Mast cells activated | Quartiles | 1.09 |
| Eosinophils | Quartiles | - |
| Neutrophils | Quartiles | 1.04 |
| Node status | Negative, Positive | 1.76 |
| Tumour size | <10mm, 10-19mm, 20-29mm, 30-49mm, 50+mm | 1.27 |
| Grade | 1,2,3 | 1.1 |
| HER2 status | Negative, Positive | 1.07 |
| Observations | 755 |  |
| Events | 240 |  |

*A statistical significance test has not yet been described for this method, however non-zero values indicate variables with an important contribution to the multi-variable model. Variables with missing estimates do not contribute to the model and correspond to coefficients of zero (hazard ratio of 1).

**Supplementary Table K: Multivariable logistic regression analysis against pathological complete response adjusted for clinical factors in ER-positive disease (complete case analysis). Selected immune cell subsets are those significantly associated with outcome in univariable analysis of ER-positive breast cancer.**

| **ER-positive (CCA)** | | | | |
| --- | --- | --- | --- | --- |
| **Variable** | **Categories** | **Odds ratio** | **95% CI** | **P-value** |
| B cells memory | Quartiles | 0.93 | 0.49-1.74 | 0.81 |
| Monocytes | Quartiles | 0.47 | 0.25-0.88 | 0.02 |
| Dendritic cells resting | Quartiles | 1.24 | 0.72-2.12 | 0.44 |
| Macrophages M1 | Quartiles | 1.16 | 0.62-2.18 | 0.65 |
| B cells naive | Quartiles | 1.51 | 0.86-2.64 | 0.15 |
| T stage | T1, T2, T3, T4 | 0.81 | 0.41-1.62 | 0.56 |
| N stage | N0, N1, N2, N3 | 0.68 | 0.35-1.30 | 0.24 |
| Grade | 1,2,3 | 3.59 | 1.18-10.9 | 0.02 |
| HER2 status | Negative, Positive | 1.75 | 0.38-8.00 | 0.47 |
| **Observations** | 284 |  |  |  |

**Supplementary Table L: Multivariable logistic regression analysis against pathological complete response adjusted for clinical factors in ER-positive disease (multiple imputation). Selected immune cell subsets are those significantly associated with outcome in univariable analysis of ER-positive breast cancer.**

| **ER-positive (Imputed M=50)** | | | | |
| --- | --- | --- | --- | --- |
| **Variable** | **Categories** | **Odds ratio** | **95% CI** | **P-value** |
| B cells memory | Quartiles | 0.86 | 0.63-1.17 | 0.33 |
| Monocytes | Quartiles | 0.67 | 0.49-0.91 | 0.01 |
| Dendritic cells resting | Quartiles | 0.83 | 0.62-1.11 | 0.21 |
| Macrophages M1 | Quartiles | 1.13 | 0.84-1.52 | 0.43 |
| B cells naive | Quartiles | 1.13 | 0.88-1.45 | 0.35 |
| T stage | T1, T2, T3, T4 | 0.9 | 0.60-1.36 | 0.63 |
| N stage | N0, N1, N2, N3 | 0.91 | 0.62-1.35 | 0.65 |
| Grade | 1,2,3 | 2.58 | 1.40-4.74 | 0.002 |
| HER2 status | Negative, Positive | 1.94 | 0.86-4.40 | 0.11 |
| **Observations** | 515 |  |  |  |

**Supplementary Table M: Multivariable logistic regression analysis against pathological complete response adjusted for clinical factors in ER-negative disease (complete case analysis). Selected immune cell subsets are those significantly associated with outcome in univariable analysis of ER-positive breast cancer.**

| **ER-negative (CCA)** | | | | |
| --- | --- | --- | --- | --- |
| **Variable** | **Categories** | **Odds ratio** | **95% CI** | **P-value** |
| B cells memory | Quartiles | 1.2 | 0.94-1.53 | 0.15 |
| Monocytes | Quartiles | 1.1 | 0.89-1.35 | 0.37 |
| Dendritic cells resting | Quartiles | 1.05 | 0.83-1.33 | 0.68 |
| Macrophages M1 | Quartiles | 1.09 | 0.87-1.38 | 0.45 |
| B cells naive | Quartiles | 1.05 | 0.84-1.31 | 0.66 |
| T stage | T1, T2, T3, T4 | 0.75 | 0.56-1.01 | 0.06 |
| N stage | N0, N1, N2, N3 | 1.31 | 0.98-1.76 | 0.07 |
| Grade | 1,2,3 | 1.75 | 0.91-3.33 | 0.091 |
| HER2 status | Negative, Positive | 1.78 | 1.01-3.13 | 0.05 |
| **Observations** | 332 |  |  |  |

**Supplementary Table N: Multivariable logistic regression analysis against pathological complete response adjusted for clinical factors in ER-negative disease (multiple imputation). Selected immune cell subsets are those significantly associated with outcome in univariable analysis of ER-positive breast cancer.**

| **ER-negative (Imputed M=50)** | | | | |
| --- | --- | --- | --- | --- |
| **Variable** | **Categories** | **Odds ratio** | **95% CI** | **P-value** |
| B cells memory | Quartiles | 1.3 | 1.08-1.56 | 0.006 |
| Monocytes | Quartiles | 0.95 | 0.81-1.11 | 0.51 |
| Dendritic cells resting | Quartiles | 1.03 | 0.87-1.22 | 0.74 |
| Macrophages M1 | Quartiles | 1.11 | 0.93-1.32 | 0.24 |
| B cells naive | Quartiles | 1.09 | 0.93-1.27 | 0.3 |
| T stage | T1, T2, T3, T4 | 0.79 | 0.61-1.02 | 0.07 |
| N stage | N0, N1, N2, N3 | 1.16 | 0.88-1.52 | 0.29 |
| Grade | 1,2,3 | 1.56 | 1.01-2.42 | 0.04 |
| HER2 status | Negative, Positive | 1.65 | 1.06-2.55 | 0.03 |
| **Observations** | 566 |  |  |  |

**Supplementary Table O: Multivariable logistic regression analysis against pathological complete response adjusted for clinical factors in ER-negative disease (complete case analysis). Selected immune cell subsets are those significantly associated with outcome in univariable analysis of ER-negative breast cancer.**

| **ER-negative(CCA)** | | | | |
| --- | --- | --- | --- | --- |
| **Variable** | **Categories** | **Odds ratio** | **95% CI** | **P-value** |
| Macrophages M2 | Quartiles | 0.75 | 0.58-0.96 | 0.02 |
| Mast cells resting | Quartiles | 0.77 | 0.61-0.97 | 0.03 |
| B cells memory | Quartiles | 1.08 | 0.86-1.36 | 0.51 |
| T cells follicular helper | Quartiles | 1.19 | 0.95-1.49 | 0.13 |
| T stage | T1, T2, T3, T4 | 0.73 | 0.54-0.99 | 0.05 |
| N stage | N0, N1, N2, N3 | 1.15 | 0.85-1.56 | 0.35 |
| Grade | 1,2,3 | 1.49 | 0.76-2.94 | 0.25 |
| HER2 status | Negative, Positive | 1.77 | 0.99-3.16 | 0.06 |
| **Observations** | 332 |  |  |  |

**Supplementary Table P: Multivariable logistic regression analysis against pathological complete response adjusted for clinical factors in ER-negative disease (multiple imputation). Selected immune cell subsets are those significantly associated with outcome in univariable analysis of ER-negative breast cancer.**

| **ER-negative (Imputed M=50)** | | | | |
| --- | --- | --- | --- | --- |
| **Variable** | **Categories** | **Odds ratio** | **95% CI** | **P-value** |
| Macrophages M2 | Quartiles | 0.83 | 0.69-1.00 | 0.05 |
| Mast cells resting | Quartiles | 0.89 | 0.76-1.06 | 0.19 |
| B cells memory | Quartiles | 1.2 | 1.01-1.42 | 0.04 |
| T cells follicular helper | Quartiles | 1.31 | 1.11-1.56 | 0.002 |
| T stage | T1, T2, T3, T4 | 0.76 | 0.59-0.99 | 0.04 |
| N stage | N0, N1, N2, N3 | 1.05 | 0.79-1.40 | 0.73 |
| Grade | 1,2,3 | 1.4 | 0.90-2.18 | 0.14 |
| HER2 status | Negative, Positive | 1.73 | 1.11-2.70 | 0.02 |
| **Observations** | 566 |  |  |  |

**Supplementary Table Q: Multivariable logistic regression analysis against pathological complete response adjusted for clinical factors in ER-positive disease (complete case analysis). Selected immune cell subsets are those significantly associated with outcome in univariable analysis of ER-negative breast cancer.**

| **ER-positive(CCA)** | | | | |
| --- | --- | --- | --- | --- |
| **Variable** | **Categories** | **Odds ratio** | **95% CI** | **P-value** |
| Macrophages M2 | Quartiles | 0.92 | 0.55-1.55 | 0.77 |
| Mast cells resting | Quartiles | 0.73 | 0.42-1.27 | 0.26 |
| B cells memory | Quartiles | 0.69 | 0.42-1.14 | 0.15 |
| T cells follicular helper | Quartiles | 1.02 | 0.63-1.65 | 0.93 |
| T stage | T1, T2, T3, T4 | 0.83 | 0.44-1.58 | 0.58 |
| N stage | N0, N1, N2, N3 | 0.61 | 0.32-1.16 | 0.13 |
| Grade | 1,2,3 | 2.57 | 0.92-7.20 | 0.07 |
| HER2 status | Negative, Positive | 1.73 | 0.41-7.33 | 0.45 |
| **Observations** | 284 |  |  |  |

**Supplementary Table R: Multivariable logistic regression analysis against pathological complete response adjusted for clinical factors in ER-positive disease (multiple imputation). Selected immune cell subsets are those significantly associated with outcome in univariable analysis of ER-negative breast cancer.**

| **ER-positive(Imputed M=50)** | | | | |
| --- | --- | --- | --- | --- |
| **Variable** | **Categories** | **Odds ratio** | **95% CI** | **P-value** |
| Macrophages M2 | Quartiles | 1.02 | 0.78-1.34 | 0.89 |
| Mast cells resting | Quartiles | 1.02 | 0.77-1.35 | 0.9 |
| B cells memory | Quartiles | 0.77 | 0.59-1.00 | 0.05 |
| T cells follicular helper | Quartiles | 0.91 | 0.70-1.19 | 0.5 |
| T stage | T1, T2, T3, T4 | 0.8 | 0.54-1.17 | 0.25 |
| N stage | N0, N1, N2, N3 | 0.94 | 0.63-1.41 | 0.78 |
| Grade | 1,2,3 | 2.57 | 1.40-4.70 | 0.002 |
| HER2 status | Negative, Positive | 1.76 | 0.80-3.89 | 0.16 |
| **Observations** | 515 |  |  |  |
