# Supplementary figures and images for: Patterns of Immune Infiltration in Breast Cancer and Their Clinical Implications: A Gene-Expression-Based Retrospective Study
Source: PLoS Med. 2016 Dec 13;13(12):e1002194. doi: 10.1371/journal.pmed.1002194 (PMC5154505; doi:10.1371/journal.pmed.1002194)

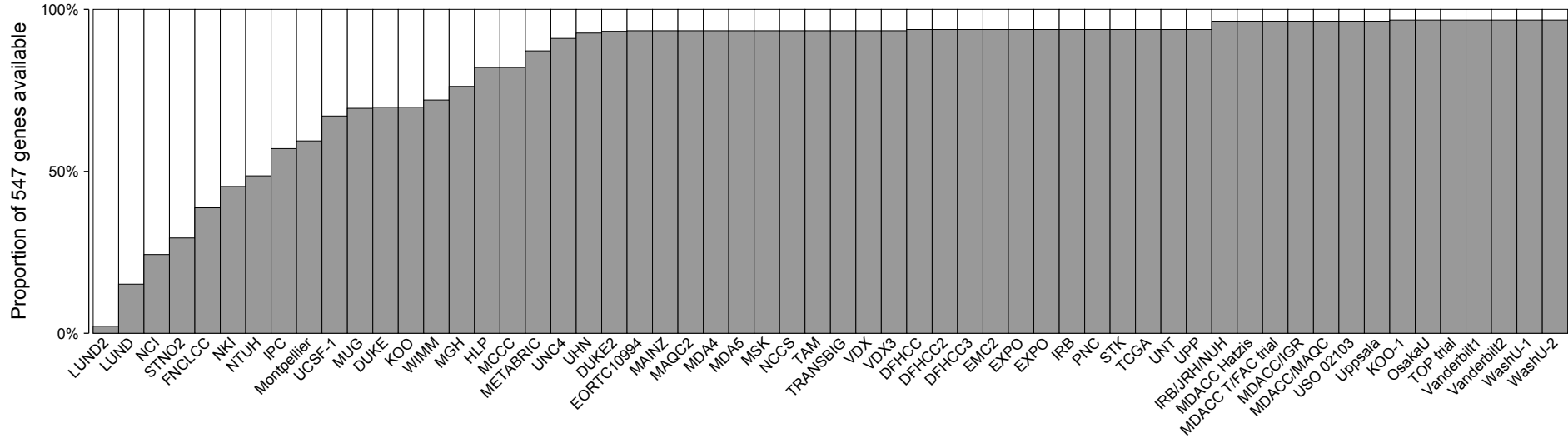

Supplement: S1 Fig — (PDF) [file pmed.1002194.s002.pdf]

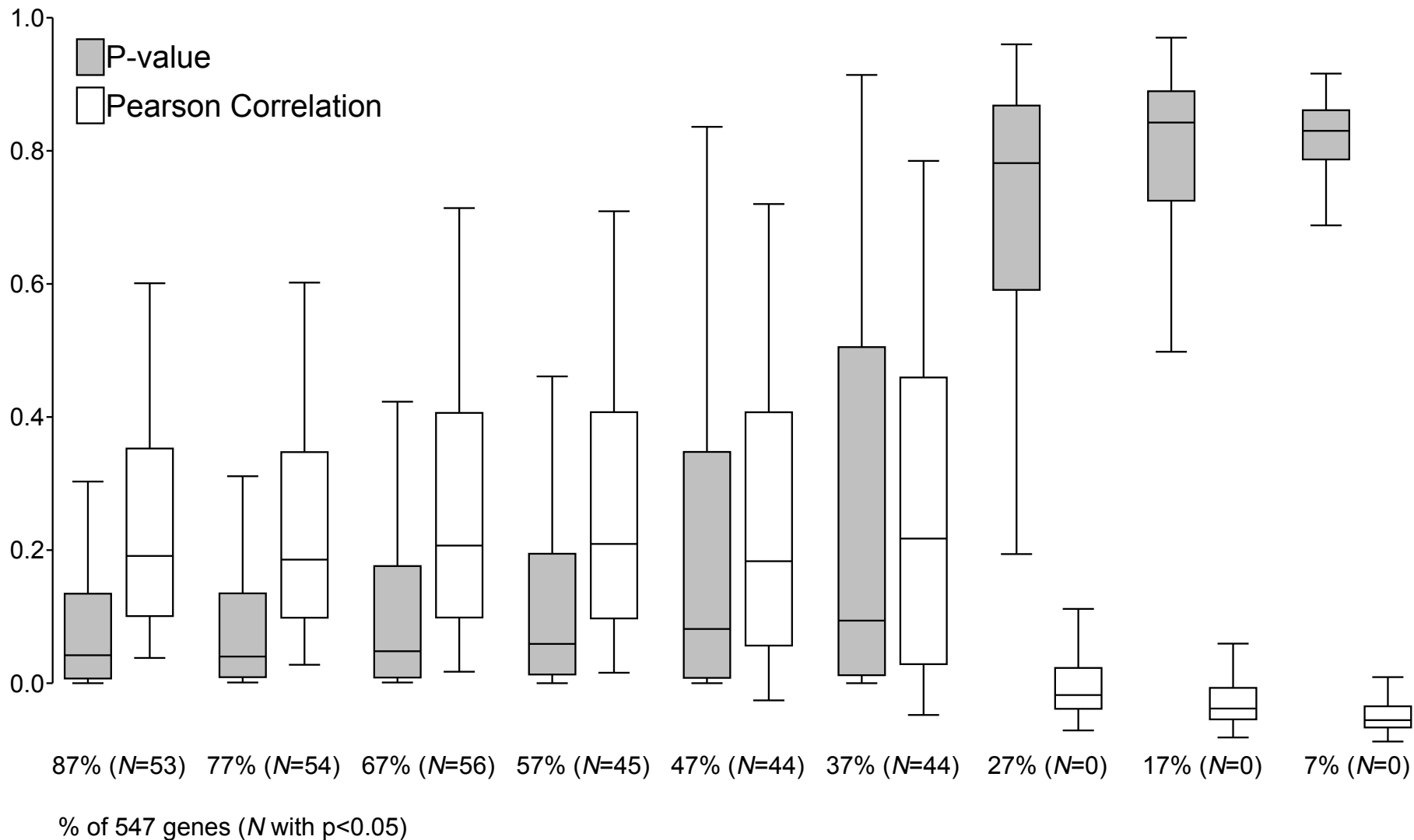

Supplement: S2 Fig — Outliers not shown. (PDF) [file pmed.1002194.s003.pdf]

% of 547 genes ( $N$  with  $p < 0.05$ )

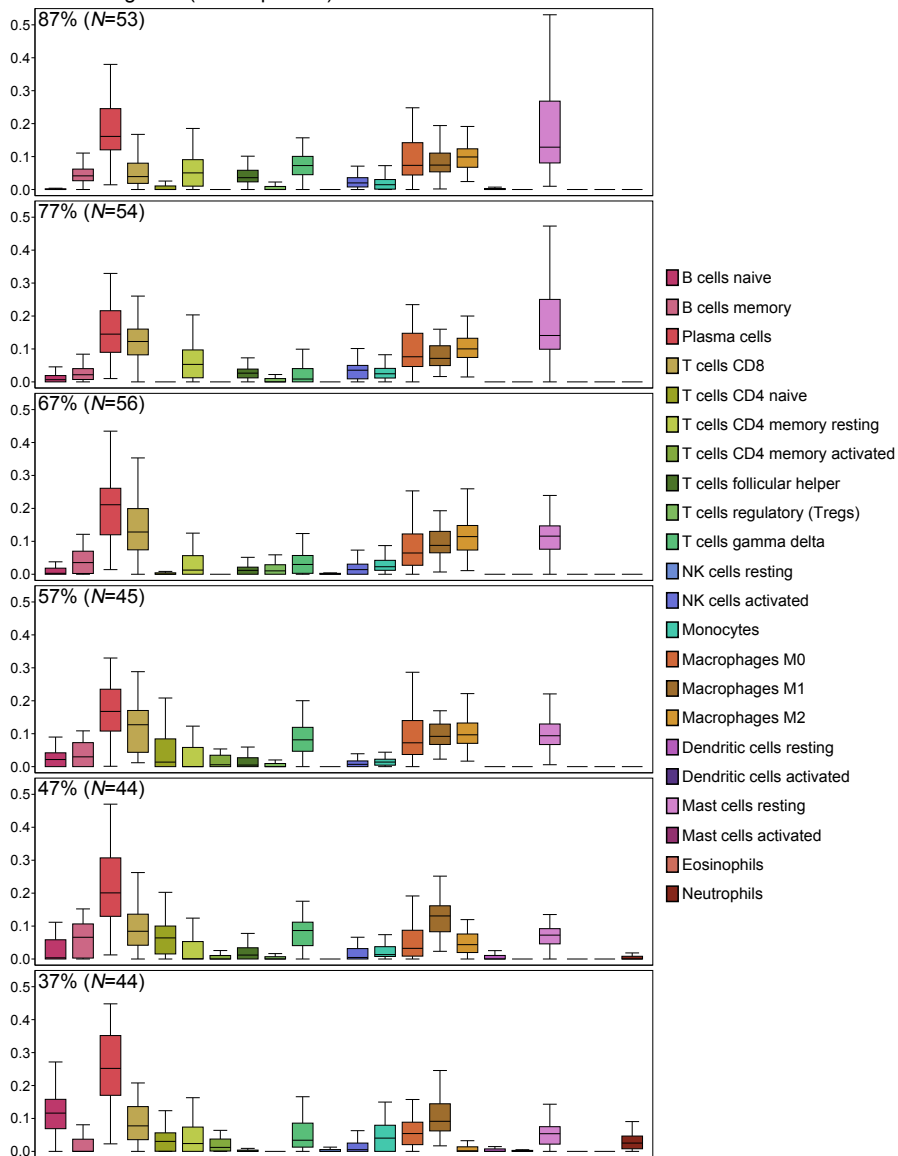

Supplement: S3 Fig — Outliers not shown. (PDF) [file pmed.1002194.s004.pdf]

ER-Positive

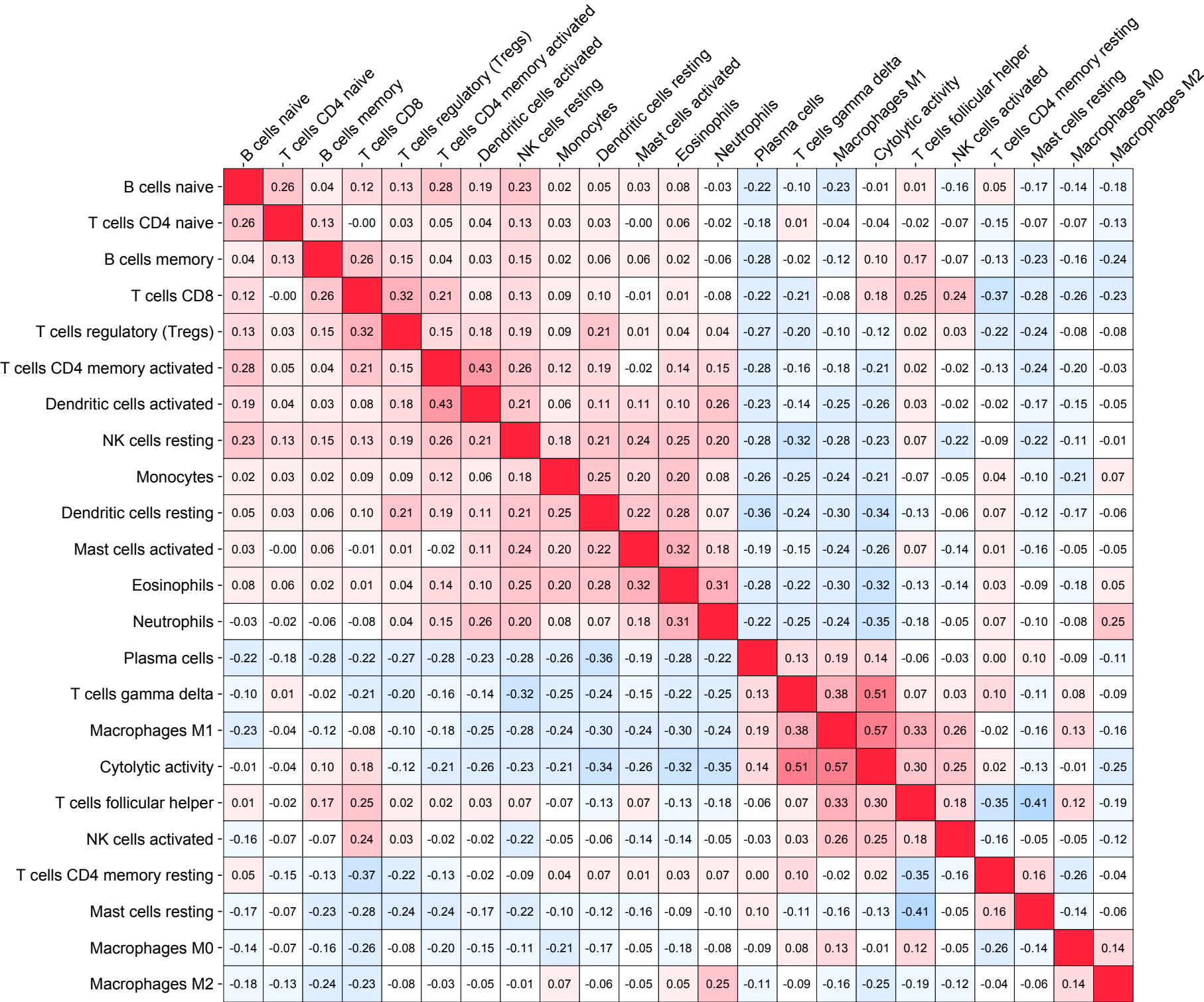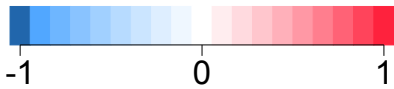

Correlation

Supplement: S4 Fig — Variables have been ordered by average linkage clustering. For comparison, cytolytic activity has been rescaled to range between zero and one separately in each study. (PDF) [file pmed.1002194.s005.pdf]

ER-Negative

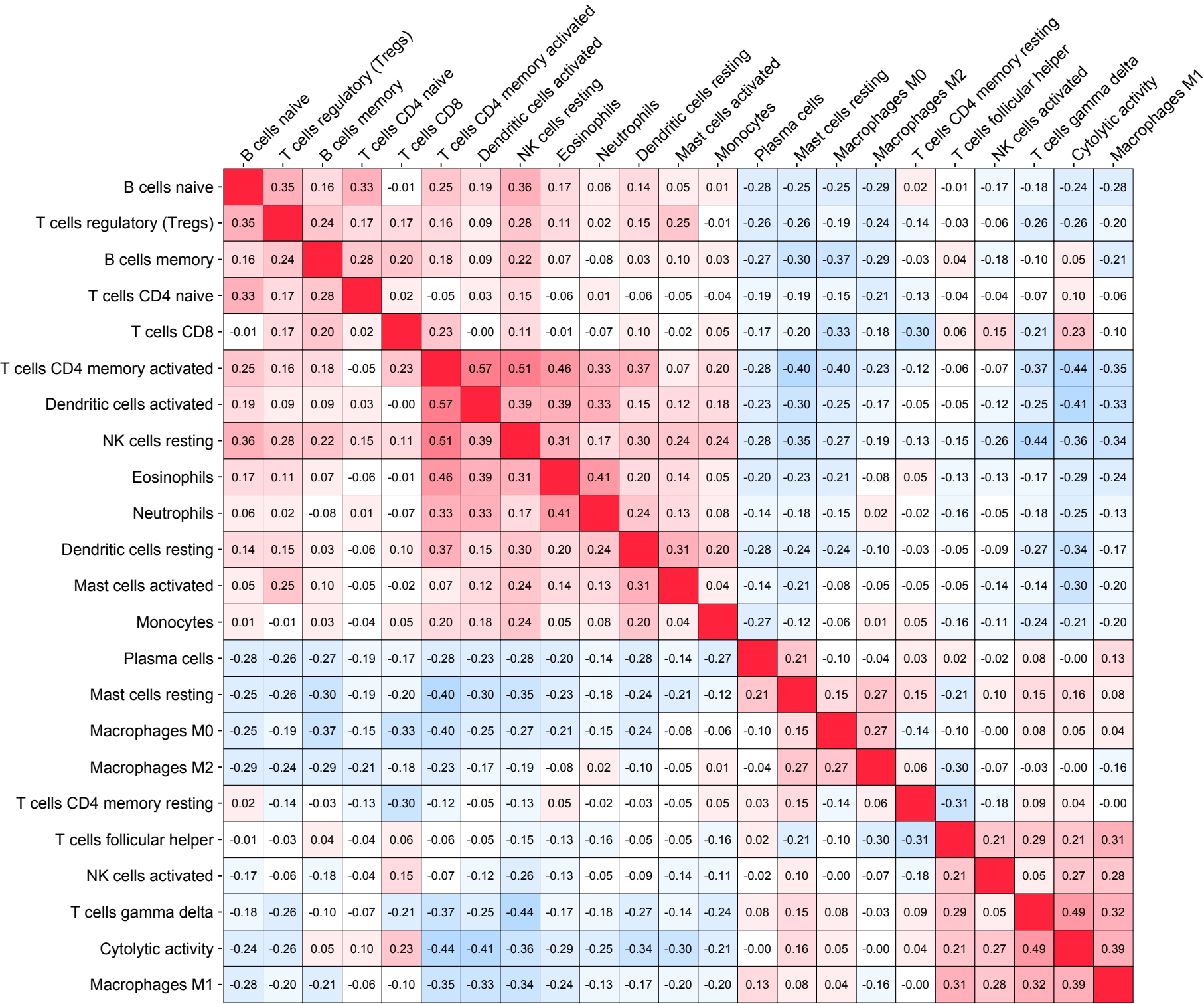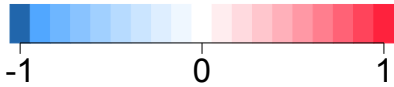

Correlation

Supplement: S5 Fig — Variables have been ordered by average linkage clustering. For comparison, cytolytic activity has been rescaled to range between zero and one separately in each study. (PDF) [file pmed.1002194.s006.pdf]

ER-Positive

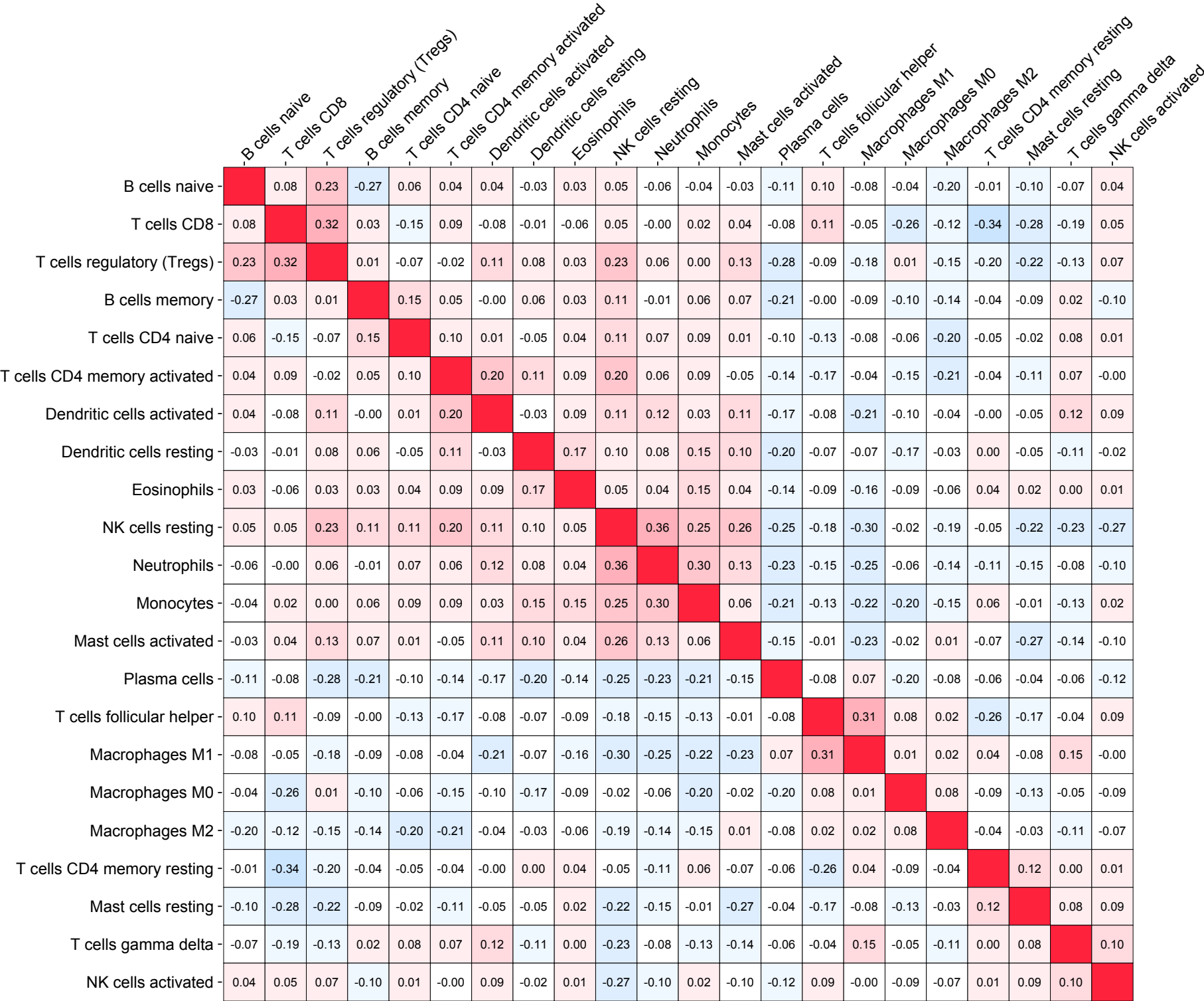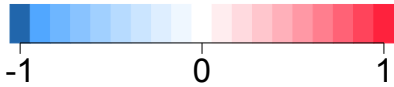

Correlation

Supplement: S6 Fig — Variables have been ordered by average linkage clustering. (PDF) [file pmed.1002194.s007.pdf]

ER-Negative

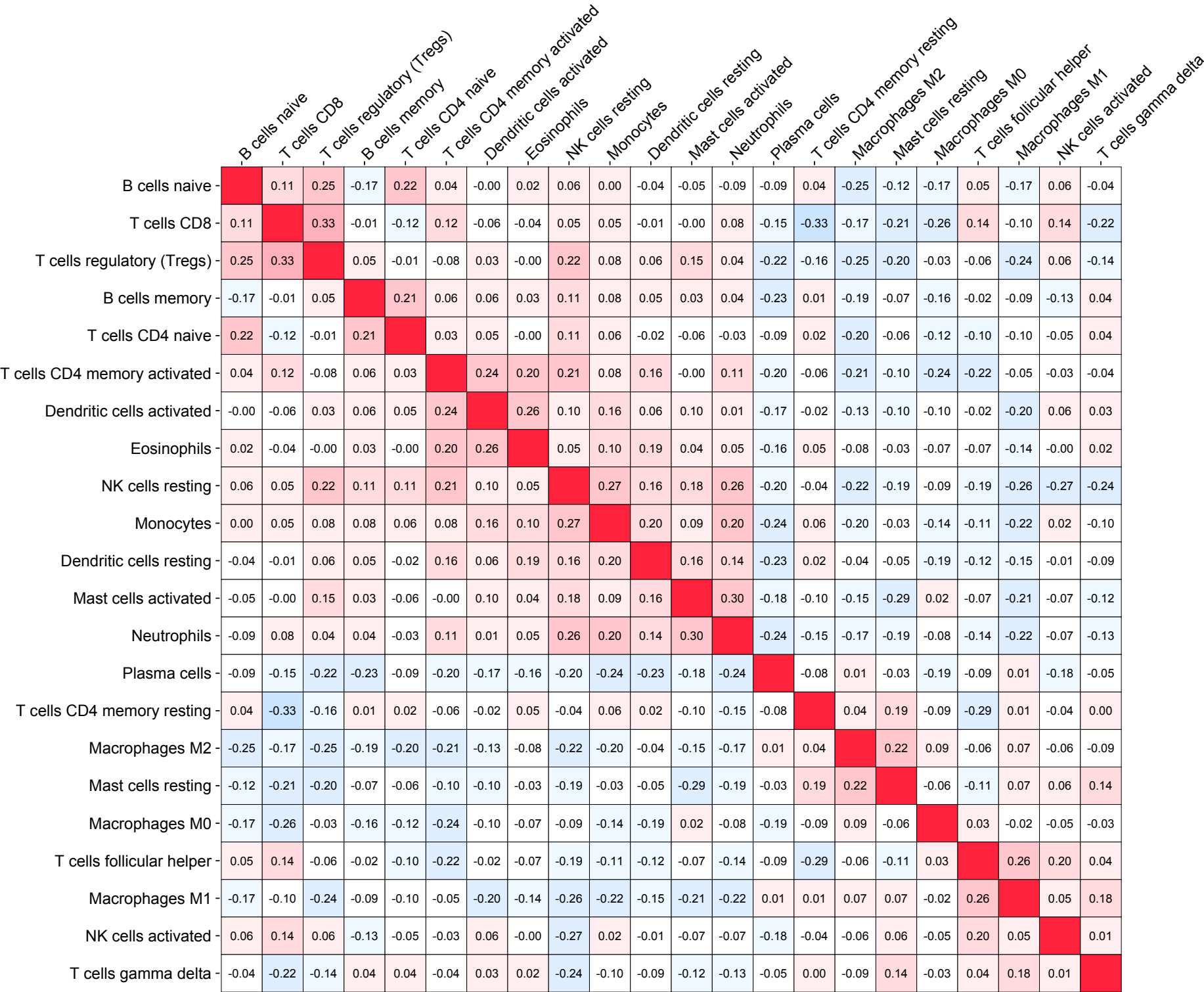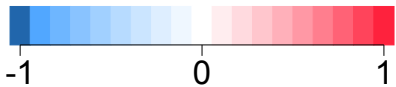

Correlation

Supplement: S7 Fig — Variables have been ordered by average linkage clustering. (PDF) [file pmed.1002194.s008.pdf]

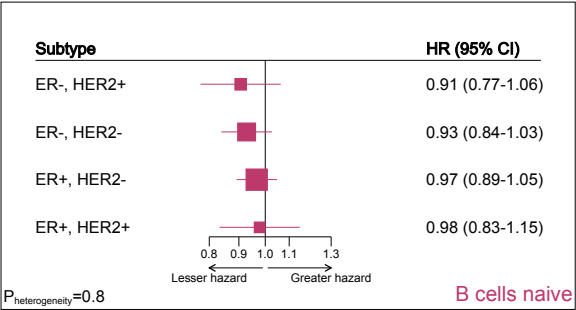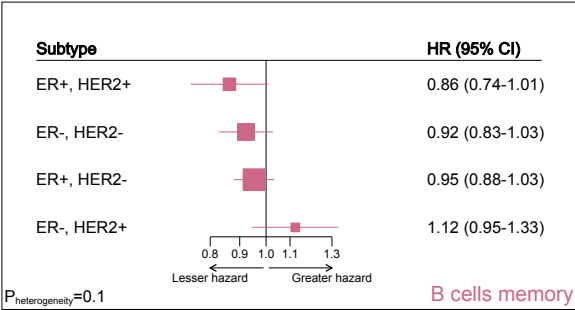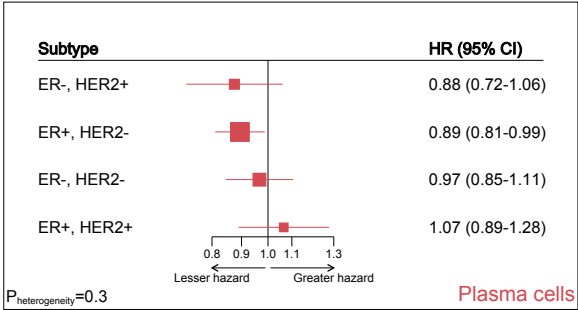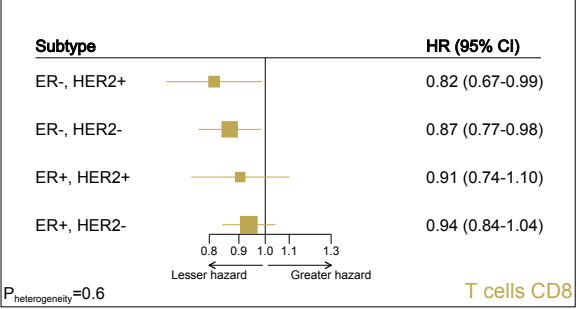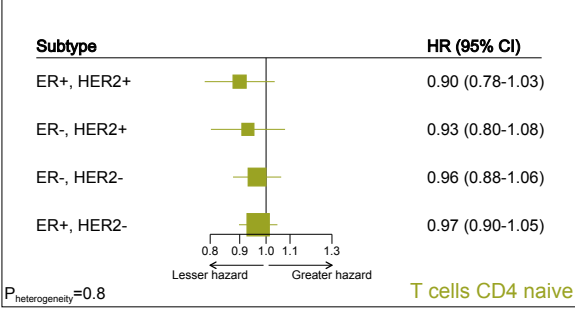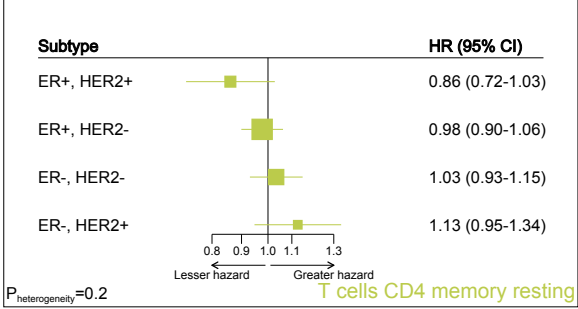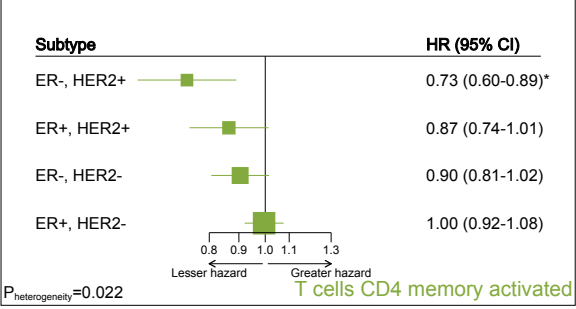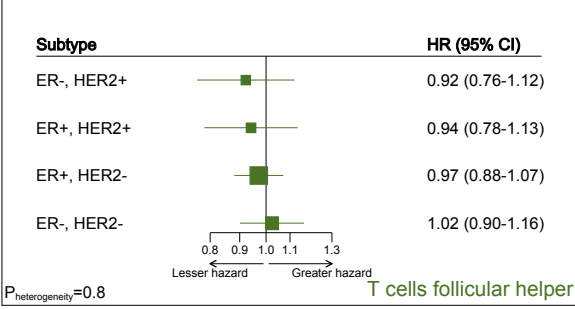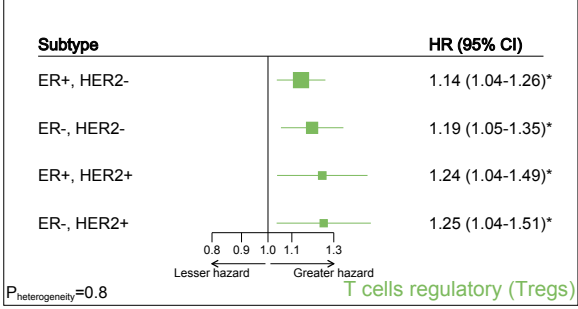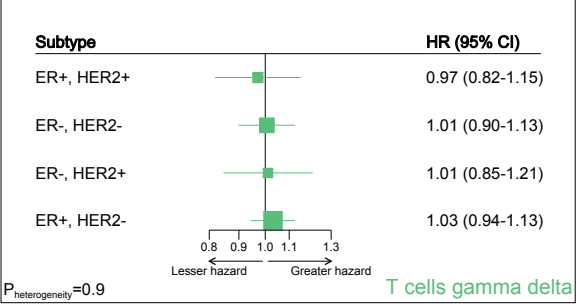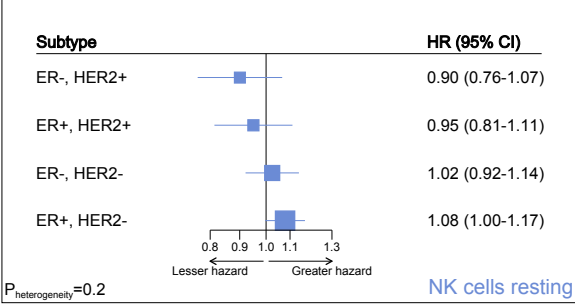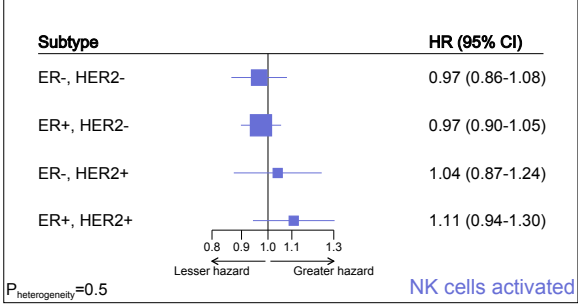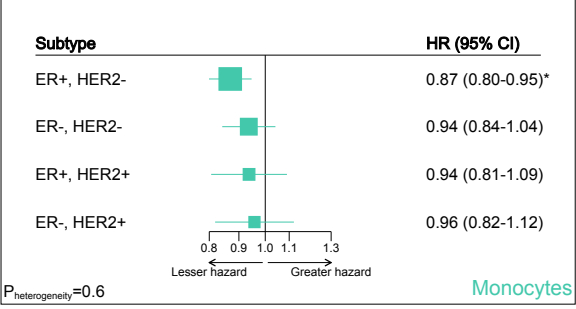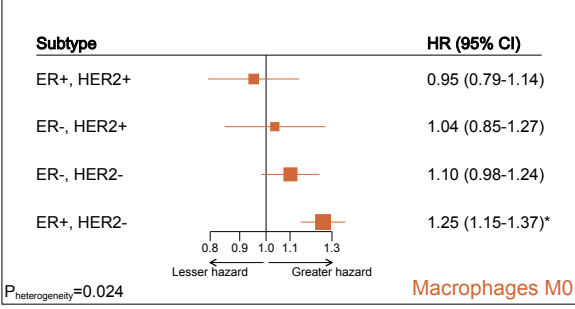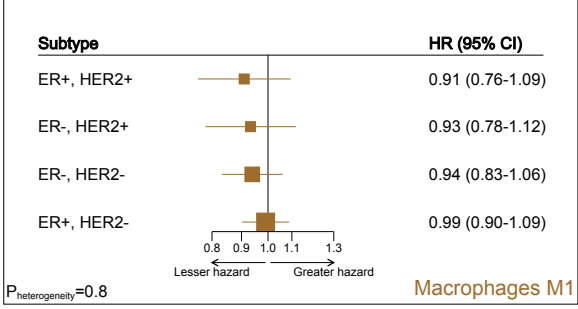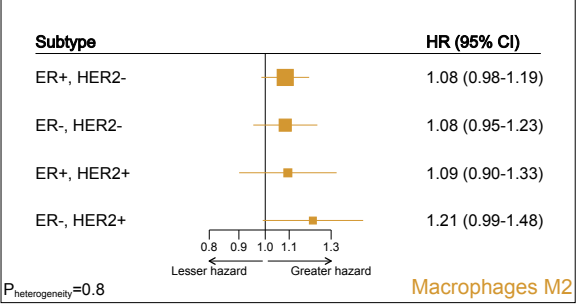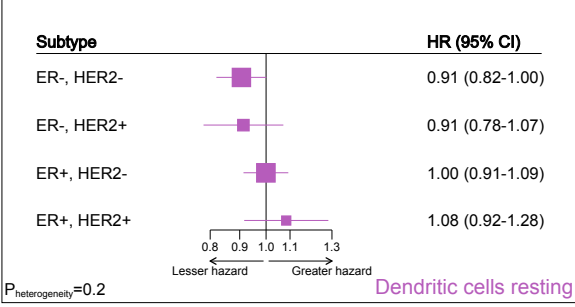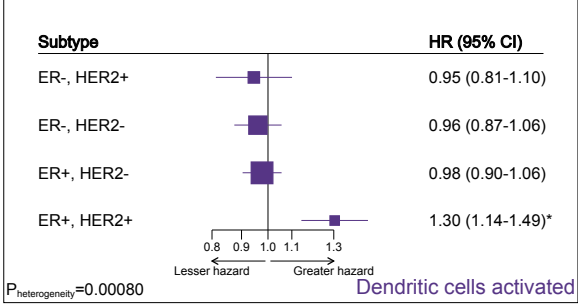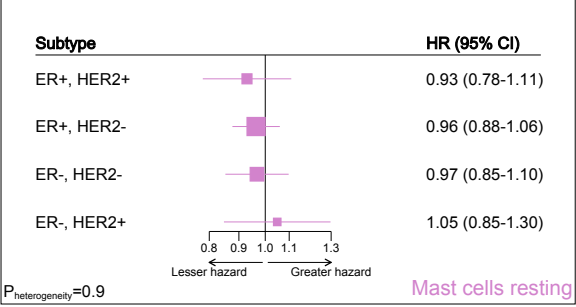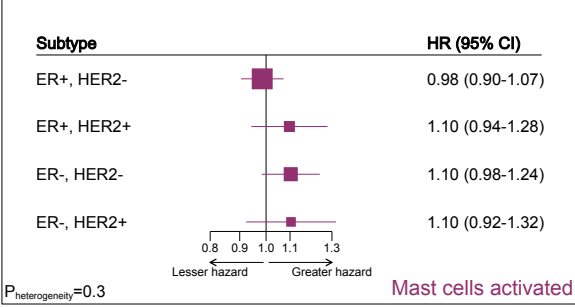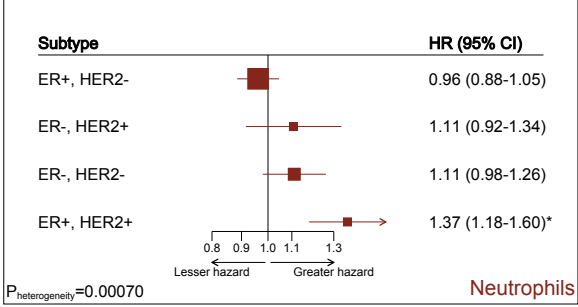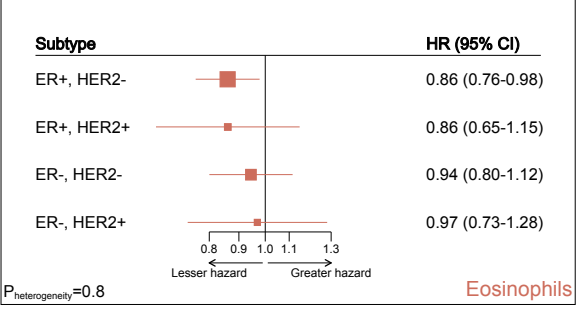

Group (subjects/events)  
ER-, HER2- (994/302)  
ER+, HER2- (2072/500)  
ER-, HER2+ (374/131)  
ER+, HER2+ (458/141)

Supplement: S8 Fig — (PDF) [file pmed.1002194.s009.pdf]

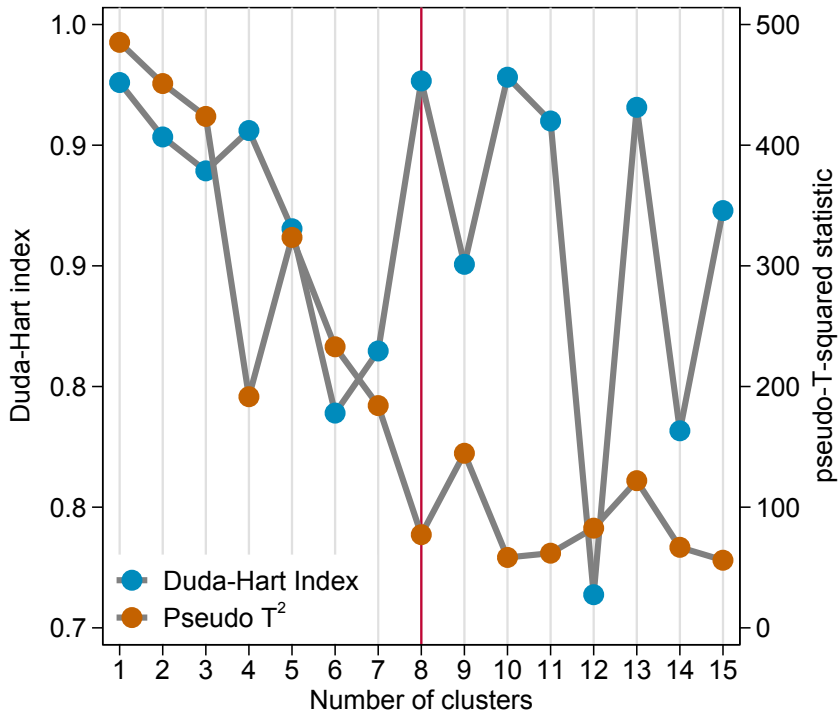

Supplement: S10 Fig — (PDF) [file pmed.1002194.s011.pdf]

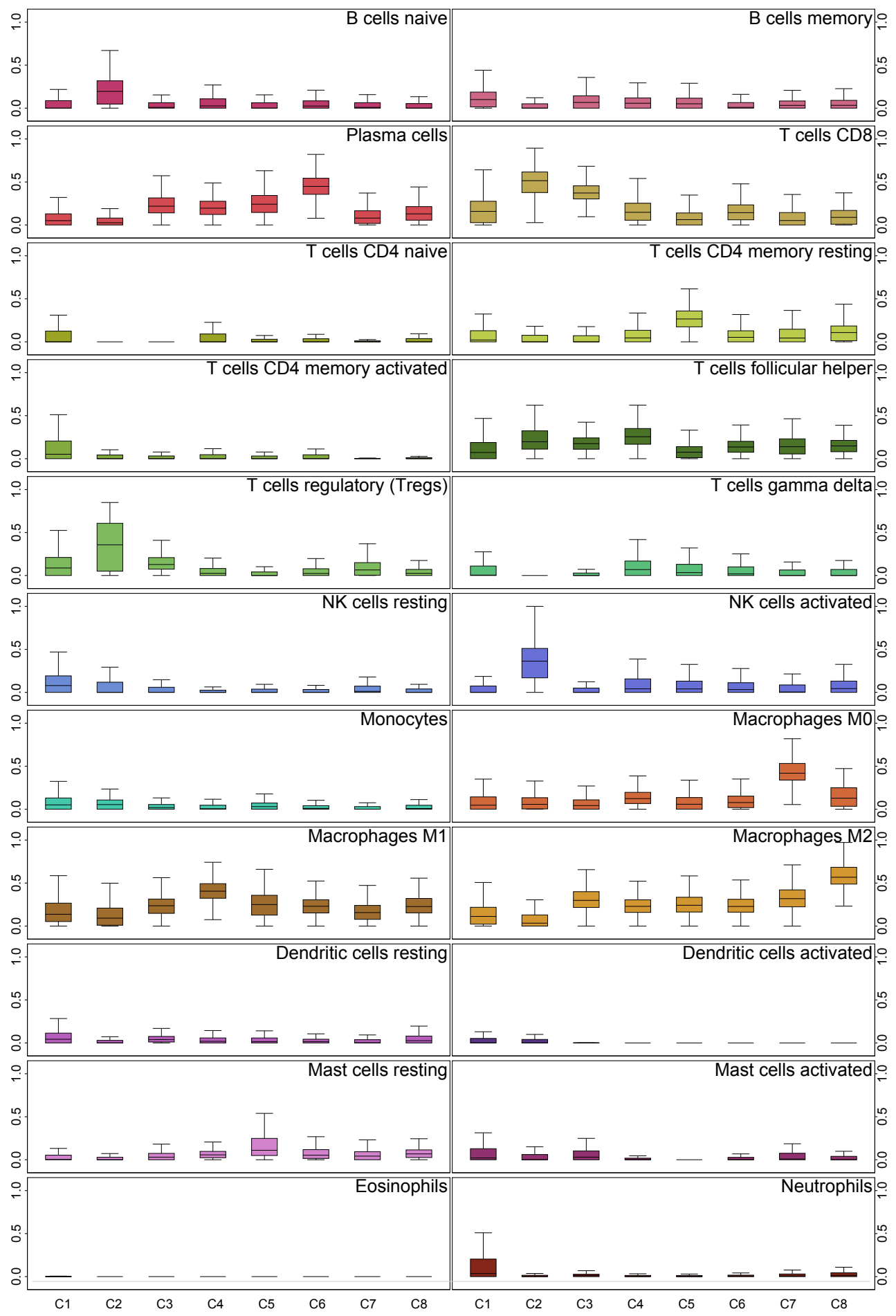

Supplement: S12 Fig — Proportions have been rescaled to range between zero and one. Outliers not shown. (PDF) [file pmed.1002194.s013.pdf]

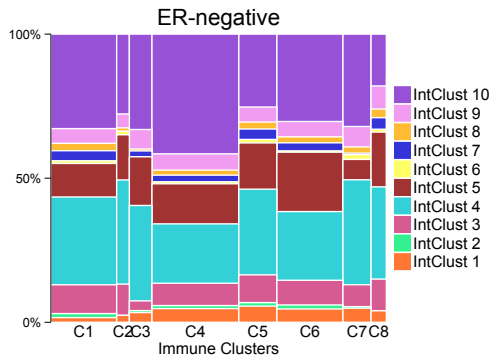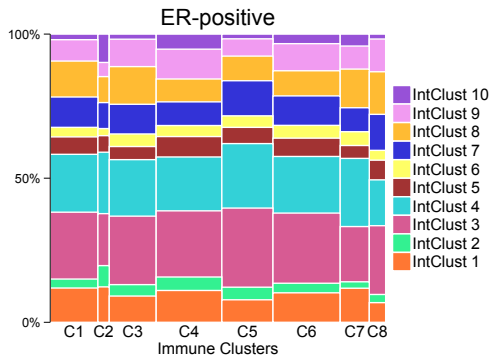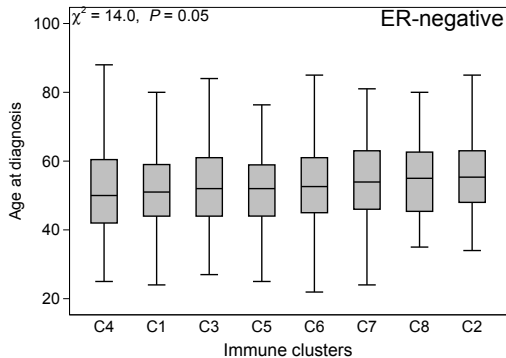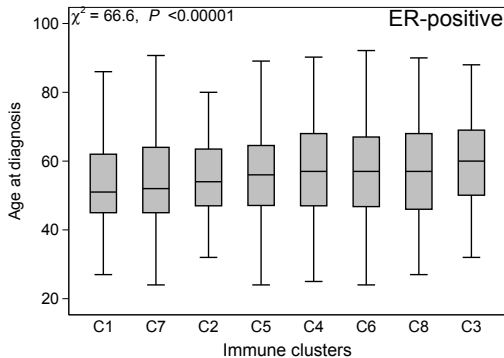

Supplement: S13 Fig — (Top panel) Spine plots of the relationship between immune cluster and IntClust subtype. (Bottom panel) Box plots of the distribution of age at diagnosis by immune cluster. Outliers are not shown. p-Values are from Kruskal-Wallis tests. (PDF) [file pmed.1002194.s014.pdf]
