# Supplementary material for: Patterns of Immune Infiltration in Breast Cancer and Their Clinical Implications: A Gene-Expression-Based Retrospective Study
Source: PLoS Med. 2016 Dec 13;13(12):e1002194. doi: 10.1371/journal.pmed.1002194 (PMC5154505; doi:10.1371/journal.pmed.1002194)
Supplement: S11 Fig — Proportions, by each cell type, have been rescaled to range between zero and one. (PDF) [file pmed.1002194.s012.pdf]

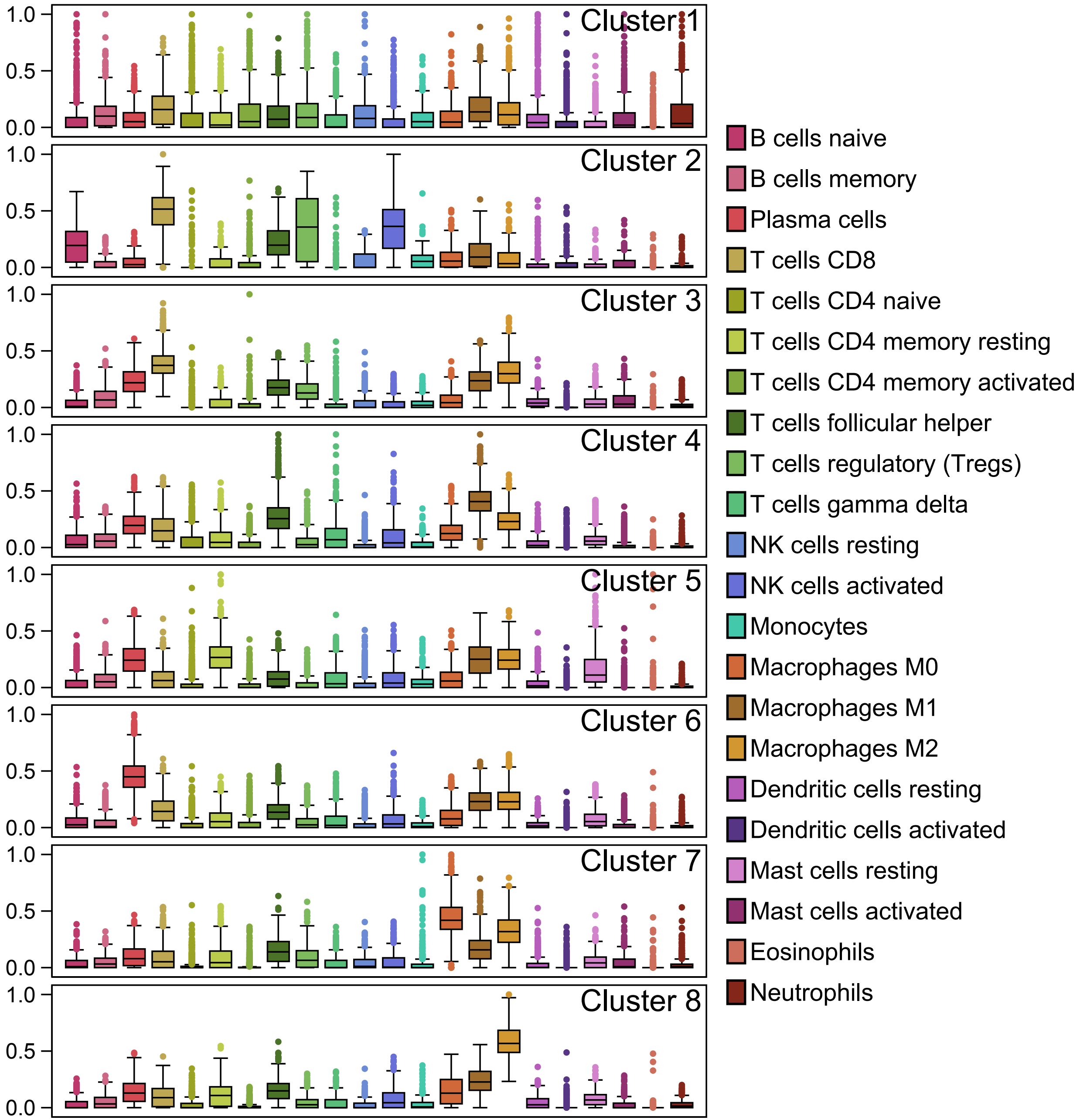

1.0
0.5
0.0
Cluster 1
Cluster 2
Cluster 3
Cluster 4
Cluster 5
Cluster 6
Cluster 7
Cluster 8
B cells naive
B cells memory
Plasma cells
T cells CD8
T cells CD4 naive
T cells CD4 memory resting
T cells CD4 memory activated
T cells follicular helper
T cells regulatory (Tregs)
T cells gamma delta
NK cells resting
NK cells activated
Monocytes
Macrophages M0
Macrophages M1
Macrophages M2
Dendritic cells resting
Dendritic cells activated
Mast cells resting
Mast cells activated
Eosinophils
Neutrophils
