# Supplementary material for: Patterns of Immune Infiltration in Breast Cancer and Their Clinical Implications: A Gene-Expression-Based Retrospective Study
Source: PLoS Med. 2016 Dec 13;13(12):e1002194. doi: 10.1371/journal.pmed.1002194 (PMC5154505; doi:10.1371/journal.pmed.1002194)
Supplement: S3 Table — (DOC) [file pmed.1002194.s017.doc]

STROBE Statement—Checklist of items that should be included in reports of ***cross-sectional studies***

|  | Item No | Recommendation |
| --- | --- | --- |
| **Title and abstract** | 1 | (*a*) Indicate the study’s design with a commonly used term in the title or the abstract  The term “Retrospective Study” is included in the title. |
|  |  | (*b*) Provide in the abstract an informative and balanced summary of what was done and what was found  Descriptive and quantitative results are provided with measures of precision. A sentence describing the limitations of the study is also included. |
| Introduction | | |
| Background/rationale | 2 | Explain the scientific background and rationale for the investigation being reported  Stated in the Introduction. |
| Objectives | 3 | State specific objectives, including any prespecified hypotheses  Study aims are described at the end of the Introduction. |
| Methods | | |
| Study design | 4 | Present key elements of study design early in the paper  Overall study design is specified at the end of the Introduction and the beginning of the methods. Figure 1 is a CONSORT-style flow diagram of each analysis stage. |
| Setting | 5 | Describe the setting, locations, and relevant dates, including periods of recruitment, exposure, follow-up, and data collection  The study uses 56 previously published studies with variable settings, recruitment periods and follow up. These are partly detailed in Supplementary Table S1 and further detailed in corresponding publications referenced in Supplementary Table S1. |
| Participants | 6 | (*a*) Give the eligibility criteria, and the sources and methods of selection of participants  Methods: Gene expression datasets |
| Variables | 7 | Clearly define all outcomes, exposures, predictors, potential confounders, and effect modifiers. Give diagnostic criteria, if applicable  Methods: Gene expression datasets and Methods: Statistical analyses and Supplementary Table S1 |
| Data sources/ measurement | 8* | For each variable of interest, give sources of data and details of methods of assessment (measurement). Describe comparability of assessment methods if there is more than one group  Methods: Gene expression datasets and Supplementary Table S1 |
| Bias | 9 | Describe any efforts to address potential sources of bias  Methods: Statistical analyses |
| Study size | 10 | Explain how the study size was arrived at  Methods: Gene expression datasets |
| Quantitative variables | 11 | Explain how quantitative variables were handled in the analyses. If applicable, describe which groupings were chosen and why  Methods: Statistical analyses |
| Statistical methods | 12 | (*a*) Describe all statistical methods, including those used to control for confounding  Methods: Statistical analyses |
|  |  | (*b*) Describe any methods used to examine subgroups and interactions  Methods: Statistical analyses |
|  |  | (*c*) Explain how missing data were addressed  Methods: Statistical analyses |
|  |  | (*d*) If applicable, describe analytical methods taking account of sampling strategy  Not applicable |
|  |  | (*e*) Describe any sensitivity analyses  Methods: Statistical analyses |
| Results | | |
| Participants | 13* | (a) Report numbers of individuals at each stage of study—eg numbers potentially eligible, examined for eligibility, confirmed eligible, included in the study, completing follow-up, and analysed  Figure 1: CSORT-style flow diagram |
|  |  | (b) Give reasons for non-participation at each stage  Figure 1: CSORT-style flow diagram |
|  |  | (c) Consider use of a flow diagram  Figure 1: CSORT-style flow diagram |
| Descriptive data | 14* | (a) Give characteristics of study participants (eg demographic, clinical, social) and information on exposures and potential confounders  Supplementary Tables S1 and S2 |
|  |  | (b) Indicate number of participants with missing data for each variable of interest  Supplementary Figure S2 |
| Outcome data | 15* | Report numbers of outcome events or summary measures  Detailed in all Figures and Tables of Cox-proportional hazards models and Logistic regression models |
| Main results | 16 | (*a*) Give unadjusted estimates and, if applicable, confounder-adjusted estimates and their precision (eg, 95% confidence interval). Make clear which confounders were adjusted for and why they were included  Methods: Statistical analyses and Results and Tables of regression models. |
|  |  | (*b*) Report category boundaries when continuous variables were categorized  Methods: Statistical analyses; quartiles were computed within subgroups. |
|  |  | (*c*) If relevant, consider translating estimates of relative risk into absolute risk for a meaningful time period  Results |
| Other analyses | 17 | Report other analyses done—eg analyses of subgroups and interactions, and sensitivity analyses  Results include details of subgroup analyses including tests for interaction and sensitivity analyses |
| Discussion | | |
| Key results | 18 | Summarise key results with reference to study objectives  Discussion |
| Limitations | 19 | Discuss limitations of the study, taking into account sources of potential bias or imprecision. Discuss both direction and magnitude of any potential bias  Discussion: a discrete paragraph details limitations |
| Interpretation | 20 | Give a cautious overall interpretation of results considering objectives, limitations, multiplicity of analyses, results from similar studies, and other relevant evidence  Discussion |
| Generalisability | 21 | Discuss the generalisability (external validity) of the study results  Discussion |
| Other information | | |
| Funding | 22 | Give the source of funding and the role of the funders for the present study and, if applicable, for the original study on which the present article is based  A funding statement is included with the published article. |

*Give information separately for exposed and unexposed groups.

**Note:** An Explanation and Elaboration article discusses each checklist item and gives methodological background and published examples of transparent reporting. The STROBE checklist is best used in conjunction with this article (freely available on the Web sites of PLoS Medicine at http://www.plosmedicine.org/, Annals of Internal Medicine at http://www.annals.org/, and Epidemiology at http://www.epidem.com/). Information on the STROBE Initiative is available at www.strobe-statement.org.
