# Supplementary material for: Patterns of Immune Infiltration in Breast Cancer and Their Clinical Implications: A Gene-Expression-Based Retrospective Study
Source: PLoS Med. 2016 Dec 13;13(12):e1002194. doi: 10.1371/journal.pmed.1002194 (PMC5154505; doi:10.1371/journal.pmed.1002194)
Supplement: S9 Fig — (PDF) [file pmed.1002194.s010.pdf]

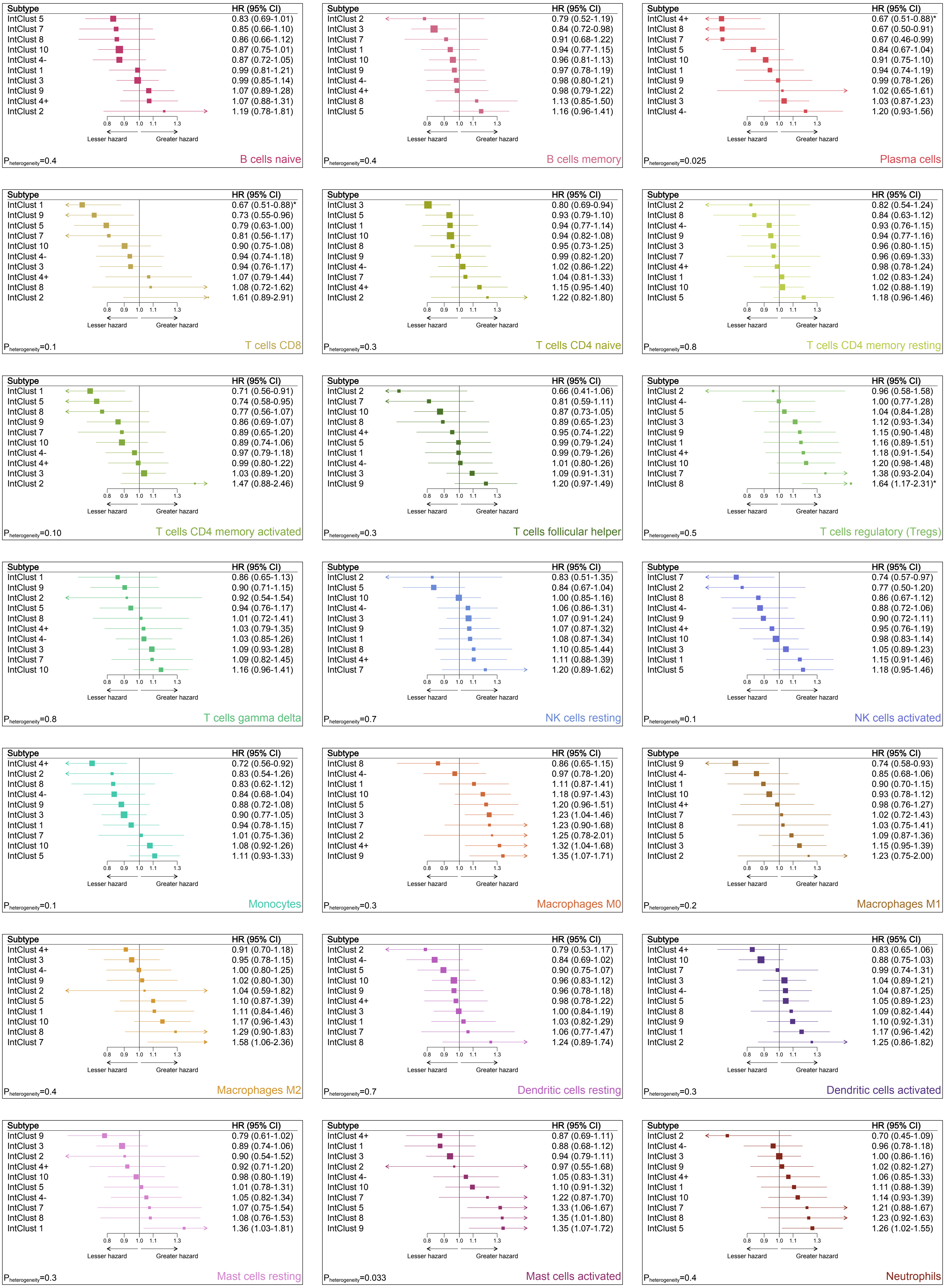

Group (subjects/events)
IntClust 1 (256/82)
IntClust 2 (86/32)
IntClust 3 (586/141)
IntClust 4- (287/94)
IntClust 4+ (439/77)
IntClust 5 (289/105)
IntClust 6 (82/22)
IntClust 7 (246/52)
IntClust 8 (238/51)
IntClust 9 (244/94)
IntClust 10 (480/137)
